# GeneiASE: Detection of condition-dependent and static allele-specific expression from RNA-seq data without haplotype information

**DOI:** 10.1038/srep21134

**Published:** 2016-02-18

**Authors:** Daniel Edsgärd, Maria Jesus Iglesias, Sarah-Jayne Reilly, Anders Hamsten, Per Tornvall, Jacob Odeberg, Olof Emanuelsson

**Affiliations:** 1 KTH Royal Institute of Technology, Science for Life Laboratory, School of Biotechnology, Division of Gene Technology, SE-171 65, Solna, Sweden; 2 Atherosclerosis Research Unit, Department of Medicine Solna, Karolinska Institutet, Center for Molecular Medicine, and Department of Cardiology, Karolinska University Hospital, Stockholm, Sweden; 3 KTH Royal Institute of Technology, Science for Life Laboratory, School of Biotechnology, Division of Proteomics, SE-171 65, Solna, Sweden; 4 Department of Clinical Science and Education, Södersjukhuset, Karolinska Institutet, Stockholm, Sweden; 5 Department of Medicine, Centre for Hematology, Karolinska University Hospital and Karolinska Institutet, Solna, Sweden

## Abstract

Allele-specific expression (ASE) is the imbalance in transcription between maternal and paternal alleles at a locus and can be probed in single individuals using massively parallel DNA sequencing technology. Assessing ASE within a single sample provides a static picture of the ASE, but the magnitude of ASE for a given transcript may vary between different biological conditions in an individual. Such condition-dependent ASE could indicate a genetic variation with a functional role in the phenotypic difference. We investigated ASE through RNA-sequencing of primary white blood cells from eight human individuals before and after the controlled induction of an inflammatory response, and detected condition-dependent and static ASE at 211 and 13021 variants, respectively. We developed a method, GeneiASE, to detect genes exhibiting static or condition-dependent ASE in single individuals. GeneiASE performed consistently over a range of read depths and ASE effect sizes, and did not require phasing of variants to estimate haplotypes. We observed condition-dependent ASE related to the inflammatory response in 19 genes, and static ASE in 1389 genes. Allele-specific expression was confirmed by validation of variants through real-time quantitative RT-PCR, with RNA-seq and RT-PCR ASE effect-size correlations r = 0.67 and r = 0.94 for static and condition-dependent ASE, respectively.

Recent advances in sequencing technologies have provided rich catalogues of genetic variation and allowed a more detailed and accurate picture of gene expression to emerge. However, the functional role of genetic variation and its impact on expression variation remains largely unknown. Genome-wide association (GWA) studies have shown that the majority of common genetic variants associated with complex diseases have a relatively modest effect and are mostly present in non-coding regions, indicating that these loci mediate their effect via cis-regulation of transcription^1^. To find cis-regulatory variants that affect transcriptional phenotypes, expression quantitative trait locus (eQTL) analysis remains a useful and common approach, attested by, e.g., the Genotype-Tissue Expression project^2^, but typically shows modest effects, requires many samples and is susceptible to inter-individual differences in expression^3^.

Another approach to identify the presence of cis-regulatory variation is to study allele-specific expression (ASE). ASE is the difference in expression between the paternal and maternal haplotype of a transcript within an individual and has been studied in humans (see references below) and other organisms, e.g., yeast^4^. An advantage of studying ASE within single individuals, as compared to eQTL analysis, is that the two alleles under study then have identical cellular environment and trans-acting factors. A detected allelic imbalance in transcription could indicate a heterozygous variant within the translated part resulting in a modified or truncated protein^5^; at a regulatory site, causing differential binding of transcription factors or epigenetic modifiers^6^,^7^,^8^,^9^,^10^; or at a splice site or UTR, affecting transcript processing^11^. Corroborating this, a study of 60 CEU HapMap individuals showed that genes exhibiting ASE are enriched for eQTLs^12^, and the ENCODE project reported correlations between allele-specific epigenetic marks and allele-specific transcription^13^. Moreover, information about ASE could be used to reduce the number of genes in the genome for which regulatory regions should be investigated in functional or genetic association studies^14^. Thus, ASE analysis is a useful approach to further our understanding of the impact of genetic variation on cellular processes, and a natural step towards a more detailed map of transcription and transcriptional regulation.

RNA sequencing (RNA-seq) enables a comparably unbiased interrogation of the transcriptional landscape, including the detection of heterozygous variants within expressed genes. Previous RNA-seq based studies of ASE have reported that roughly 20% of heterozygous variants in coding regions of the human genome display ASE^6^,^12^,^15^ and this is also the approximate prevalence in microarray-based ASE investigations^16^,^17^,^18^. There is a span from 3–5% of SNPs with ASE^9^,^19^ to 45% of genes with ASE, where the highest estimates are from cell lines or cancer cells^11^. In fact, most ASE studies have been performed on cancer cell lines^6^,^9^,^12^,^20^ with only a few exceptions^21^,^22^.

These ASE prevalence estimates pertain to the ASE that is detected between the two variants of a heterozygous allele in a single sample under a certain unchanging condition (controlled or observed). Thus, we refer to this phenomenon of ASE at a single condition, as static ASE (Fig. 1). One potential problem with assessing static ASE is the read mapping which will be biased towards preferably mapping reads with alleles identical to the reference genome thus inflating the number of false positive ASE calls. A number of methods have been suggested to counter the mapping bias^9^,^12^,^20^,^23^, collectively highlighting the importance of estimating the performance of the methods, in particular false discovery rates (FDRs), through data simulations and experimental validation. Current methods to simulate RNA-seq data for FDR estimation in ASE analysis^9^ allow at most one sequence variant per read, thus significantly underestimating FDRs, as in Heap *et al.*^19^ where simulated FDR was 1% and observed FDR 63%.

An emerging field is the investigation of the potentially differential ASE between individuals or between tissues or biological conditions within a single individual^24^. In yeast, the interplay of strain and condition effects from different growth media (glucose or ethanol) was investigated by Smith and Kruglyak using crosses of two different yeast strains^25^. Two recent microarray-based studies in human support the notion that there is a connection between treatment and ASE: Adoue *et al.*^26^ used TNF-alpha treatment to induce immune response in six HapMap samples and reported a number of transcripts that were allelically regulated by NF-kappa-B. Fairfax *et al.*^27^ investigated primary monocytes from 432 individuals and reported that more than half of cis- and many trans-eQTLs were induced in treated samples.

RNA-seq based identification of differential ASE in tumour samples and cancer cell lines was presented in a recent paper, which also provided an ASE analysis tool^22^. The authors investigated 32 samples (7 matched tumour/normal tissues and 18 cancer cell lines) and reported higher rates of static ASE in tumour samples (9–26%) as compared to normal tissue samples (0.5–2%), and that a variable fraction of genes with static ASE exhibited differential ASE (3–32% for normal tissues). In Chen *et al.*^21^, the differential ASE in a single human subject was measured by contrasting the ASE at a single time point against the ASE at several other time points. Like Mayba *et al.*^22^, this was an observational study and the subject was not in a controlled environment. Their ASE detection relied on obtaining the subject’s genomic sequence, and the authors were able to phase most variants since also the genome of the subject’s mother was sequenced. Phasing (haplotype estimation) enables the use of certain tools to analyze ASE^6^,^15^, but requires a substantially increased sequencing and analysis effort.

We refer to the phenomenon of ASE that changes between conditions at a locus (variant or gene) as condition-dependent ASE (cd-ASE), or individual condition-dependent ASE (icd-ASE) when data are from two conditions within a single individual (Fig. 1). We used RNA-seq data to study condition-dependent and static ASE in human primary white blood cells from eight individuals with and without treatment by the inflammatory stimulus lipopolysaccharide (LPS). We applied methods to detect ASE at single nucleotide variants (SNVs), and we developed a novel method, GeneiASE, to detect condition-dependent and static ASE for genes in single individuals (Supplementary Fig. S1). GeneiASE uses RNA-seq data alone, does not need known or estimated haplotypes, and is available as a downloadable software package. We evaluated our methods over a range of read depths and effect sizes, and we confirmed the presence of both condition-dependent and static ASE through validation by real-time quantitative RT-PCR.

## Results

### RNA-seq yielded average exome depths ranging from 66x to 358x

We RNA-sequenced 16 samples of primary white blood cells derived from LPS-treated and untreated cells from eight healthy individuals (Table 1). Reads were paired-end 2 × 100, mapped to the human reference genome (hg19) and quantified as described in Methods. Average coverage across the exome, defined as the consensus coding DNA sequences (CCDS), ranged from 66 to 358 per sample (median 164; Table 1), and the percentage of a sample’s exome covered with at least depth 10 ranged from 55% to 66% (Supplementary Fig. S2). The differential gene expression was investigated and out of 35,215 Ensembl genes, 5,395 (15.3%) were significantly differentially expressed (adjusted P < 0.05). In a principal component analysis of FPKM values (Fragments Per Kilobase of exon per Million fragments mapped), all 16 samples clustered in agreement with their condition (treated/untreated), apart from one of the LPS treated samples which showed a tendency of being an outlier (Supplementary Fig. S3). The relationship between sequencing depth and statistical power is discussed in Supplementary Methods.

### Variant calling from RNA-seq and SNP-array data

Variant calling from RNA-seq data resulted in 134,661 unique heterozygous SNVs across all 16 samples, and SNP-array genotyping resulted in 49,351 variants, requiring RNA-seq coverage at depth 10 or greater and presence in dbSNP (Supplementary Fig. S4). This yielded in total 150,887 variants accessible for ASE analysis (Table 1). The concordance between heterozygous SNV calls from RNA-seq and SNP-array was >85% for every sample (Supplementary Table S1). The allele frequency spectrum is shown in Supplementary Fig. S5.

### Detection of variants exhibiting ASE

We used Fisher’s exact test to identify individual condition-dependent ASE (icd-ASE) variants in each of the 8 individuals. Notably, this analysis is robust to mapping biases since it constitutes a comparison between conditions within the same individual. We detected 10 to 55 variants within each individual (median = 27.5) that exhibited significantly altered ASE (multiple testing corrected P < 0.05), corresponding to 0.1–0.2% of the total number of heterozygous variants within each individual (Table 1). The number of detected variants correlated to some extent with the minimum number of reads for the two samples (T, U) from an individual (Pearson’s r = 0.51). In total, 211 unique significant icd-ASE variants were detected among all individuals, 191 of which were within annotated RefSeq genes: 177 exonic, 14 intronic, 15 intergenic, and 5 in non-coding RNA. Forty-seven icd-ASE variants were non-synonymous, causing a potentially different protein variant to be expressed (Supplementary Table S2).

Eleven out of the 211 icd-ASE variants were detected as significant in two or more of the 8 individuals (5.2%). We investigated the impact of varying read depth on the false negative rate by using sustained effect size across individuals instead of demanding significant icd-ASE. We set an icd-ASE effect size threshold |ΔASE_RNA-seq_(T-U)| > 0.61, corresponding to the 90th percentile of the observed icd-ASE magnitudes among the 211 variants with significant icd-ASE. 51 variants (24%) were observed to have an icd-ASE effect size of at least this magnitude in two or more individuals (Supplementary Fig. S6). We estimated the FDR of our Fisher’s test method to 8%, by simulating allele-specific RNA-seq data allowing for multiple variants within a read (see Methods). The FDR strongly depended on the read depth: at lower depth (<30 summed over both conditions) the estimated FDR reached 30%, while gradually decreasing to about 5% at read depths higher than 500. The FDR was relatively stable over a wide range of effect sizes (Supplementary Fig. S7).

We investigated whether the change of ASE was consistent across the variants within a gene. For each of the 68 genes with at least two heterozygous variants in the same individual and at least one significant icd-ASE variant, we calculated the icd-ASE signal-to-noise ratio (SNR), defined as the average of |ΔASE_RNA-seq_(T-U)| for the variants within the gene, divided with the standard deviation (Supplementary Table S3). Forty-six genes (68%) had a SNR ≥ 2 in at least one individual, indicating a consistent icd-ASE throughout the gene (Supplementary Fig. S4).

We used a modified binomial test to identify all single variants exhibiting significant static ASE in each of the 16 samples. The modification is a means to reduce mapping-bias^12^, and static refers to the fact that the ASE is measured at a single, unchanging (and hence, static) condition. For each sample, 3.3–6.3% of the variants exhibited statistically significant static ASE (Table 1). This resulted in a total of 13,021 significant static ASE variants across all samples (8.6% of all 150,887 variants) with an estimated FDR of 3%.

### GeneiASE: a novel method to detect genes with ASE from RNA-seq data

We constructed a method capable of detecting individual condition dependent-ASE (icd-ASE), as well as static ASE, in genes from RNA-seq data without the need for haplotype estimation (phasing). We based our method on six key properties that a well-powered model should incorporate to identify genes with cd-ASE or static ASE: paired data model, beta-binomial model, random effect model, variance stabilization, proper null model estimation, and undirected effect model. These properties and their presence in some ASE detection methods are discussed in Supplementary Methods and Supplementary Table S4.

**Figure 1:**
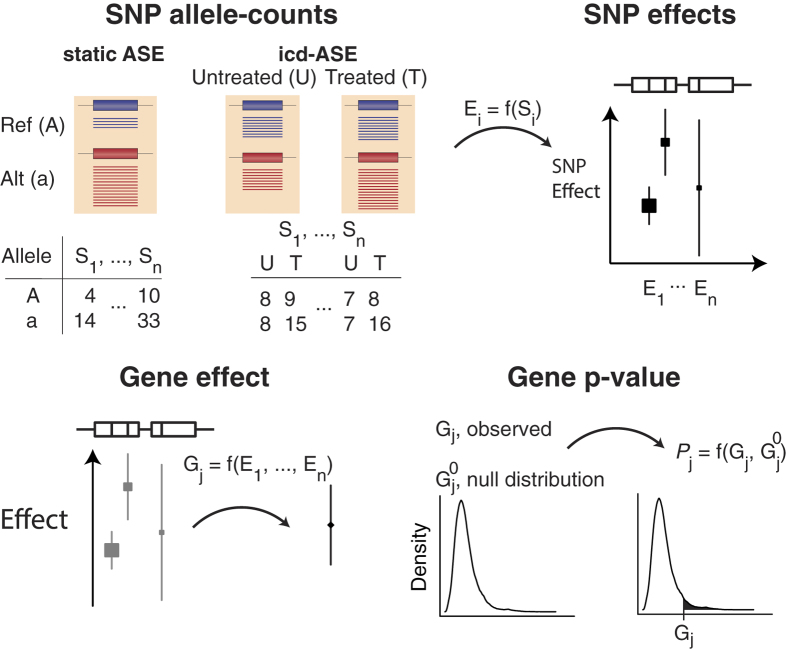
GeneiASE overview. For each SNP (**S**_i_), allele-counts are input as either a 2 × 1 table (static ASE) or a 2 × 2 table containing counts from each of two conditions (icd-ASE). Allele-counts are then converted to a test statistic for each SNP, reflecting a SNP-wise effect size (**E**_**i**_). SNP-effects are then combined via meta-analysis into a gene effect (**G**_**j**_). Finally, a p-value (**P**_**j**_) is obtained via a resampling-based null distribution (**G**_**j**_^**0**^). Red and blue thick bars represent reference (Ref, A) and alternative (Alt, a) alleles, respectively, and flanking thin black lines represent adjacent genomic DNA. Thin lines represent reads mapped to the corresponding alleles. Allele-count tables indicate reads mapped to the reference and alternative allele at different SNPs. Effects are shown as forest plots where the size of the square and length of the vertical line indicate confidence of the effect-size estimate. Upper bars in the effect-size sub-figures indicate a gene-model with two exons and with vertical lines indicating SNP positions. See Methods and Fig. S1 for more details.

Our method (Fig. 1 and Suppl. Fig. S1) used a beta-binomial null model for single variants, estimated from DNA data. For icd-ASE, a test-statistic for each variant in an individual was obtained based on the read counts for each allele in both conditions (2 × 2 table), and for static ASE from the two alleles in a single condition (2 × 1 table). The test-statistics from all variants within a gene were then combined to form a gene-wise measure. We named the method GeneiASE, reflecting that it provided a gene-based analysis of allele-specific expression for an individual. It did not, however, determine the actual haplo-isoforms. GeneiASE is available at http://geneiase.sourceforge.net.

**Figure 2:**
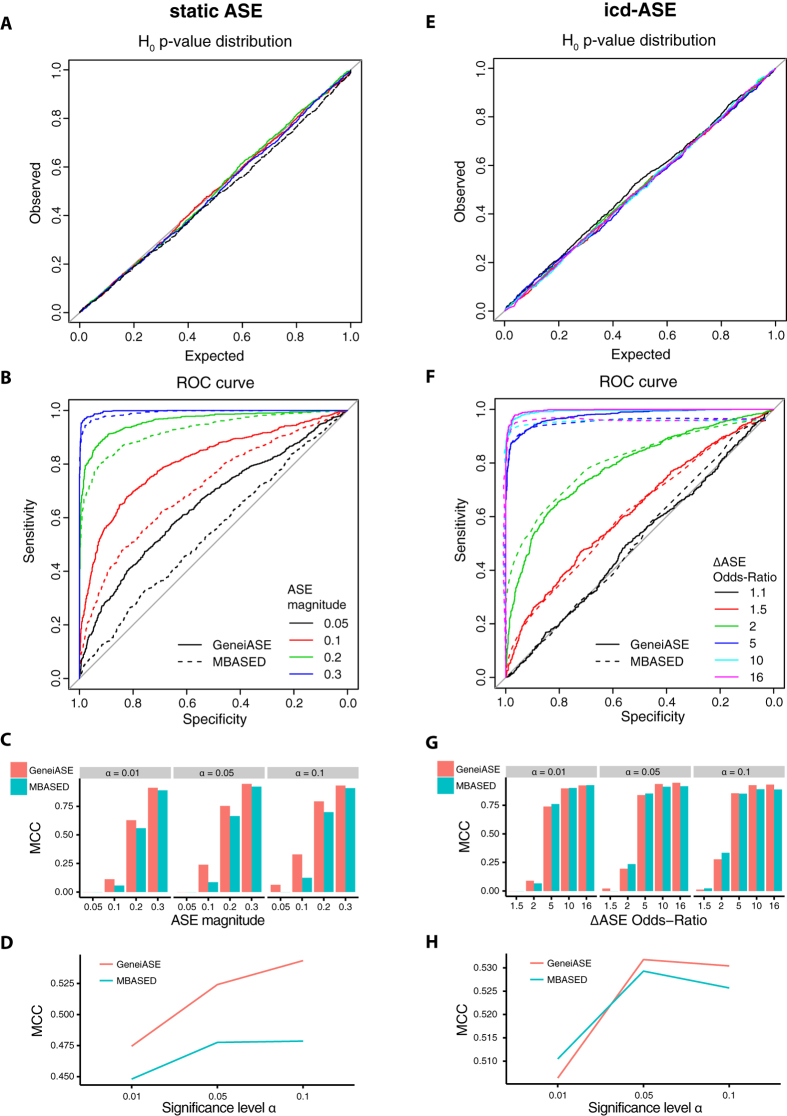
GeneiASE performance. Left column figures (**A–D**) relate to static ASE and right column figures (**E–H**) to icd-ASE. (**A,E**) show that the p-value distributions of GeneiASE are uniform under the null hypothesis of no static ASE and no icd-ASE, respectively, implying that observed FDRs reflect expected FDRs. Colors in (**A,E**) indicate simulation replicates. (**B,F**) ROC curves show that GeneiASE outperforms MBASED for static ASE and that they are even for icd-ASE. Read depths and noise level used were from sampling and estimation from empirical data (Methods). Color indicates effect size and the legend specifies level of static ASE (**B**) and icd-ASE (**F**). (**C**,**G**) show the performance of GeneiASE and MBASED for static ASE and icd-ASE, respectively, in terms of Matthew’s correlation coefficient (MCC) at different significance level cutoffs (α). (**D**,**H**) show the performance of GeneiASE and MBASED on genes across all sampled effect sizes for static ASE and icd-ASE, respectively. Values 0.05-0.3 indicate level of static ASE (**B,C**), defined in Eq. 1, and legend with values 1.1–16 indicates the odds-ratio of icd-ASE (**F,G**), defined in Eq. 2. 2000 genes were sampled from each effect size (**B–D**, **F–H**).

We calibrated GeneiASE using genomic DNA, ensuring that the test was neither overly optimistic nor too conservative. This parameter estimation adjusted for technical variability, which in addition to statistical sampling effects, yielded fluctuations around the theoretically expected 50:50 allele count ratios. GeneiASE proved to be well-calibrated as indicated by the uniform p-value distribution under the null hypothesis (Fig. 2A,E), and it was also robust under a wide range of ASE magnitudes, read depths, and noise levels using data simulated under the null hypothesis (Supplementary Figs S8 and S9).

We assessed the performance of GeneiASE on simulated data by receiver operating characteristic (ROC) curves and power analysis, and compared it to MBASED^22^, as well as an approach where we directly combined the SNV test-statistic from a modified binomial test or Fisher’s test using Stouffer’s method (Supplementary Methods). For static ASE, GeneiASE outperformed both MBASED and the modified binomial test combined with Stouffer’s method (Fig. 2B–D and Suppl. Fig. S10). For cd-ASE, GeneiASE outperformed Fisher’s test combined with Stouffer’s method (Supplementary Fig. S11), while it performed on par with MBASED in the ROC analysis (Fig. 2F), but better at commonly used significance levels (α = 0.05 and α = 0.1) using a data set with mixed effect sizes (Fig. 2G–H). Power analysis (FDR = 5%, Benjamini-Hochberg corrected) for icd-ASE with a noise level estimated from genomic DNA data showed very low power for a read depth of 10, reaching a power of 10% first at an effect size (odds-ratio) of 10. In contrast, for a read depth of 50 the power was over 60% at an odds-ratio of 10. Increasing the read depth to 100 had a minimal effect (Supplementary Fig. S12). We noted that for noise levels from zero up to that observed in the DNA data (0.22), the power was relatively strongly affected for odds ratios below five, but marginal for higher effect sizes. Corresponding power analyses for static ASE are shown in Supplementary Fig. S12.

### GeneiASE revealed 19 genes with cd-ASE and 1389 with static ASE

**Figure 3:**
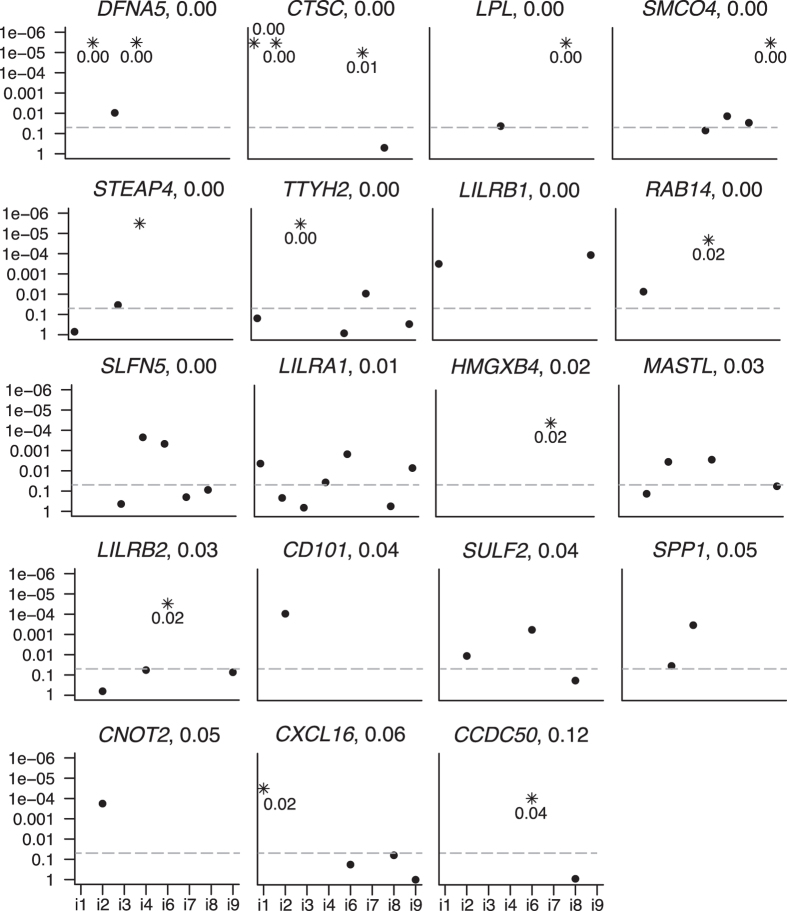
Genes with condition-dependent ASE. GeneiASE p-values for each individual (i1 – i9) for all genes that exhibited significant cd-ASE in at least one individual or in the meta-analysis. Each figure panel corresponds to one gene, with the gene name stated above each panel. Genes are ranked from left to right, top to bottom, according to their multiple testing adjusted p-value from the meta-analysis. This p-value is indicated next to the gene name. Dashed grey line indicates nominal GeneiASE p-value 0.05. A missing dot means that the gene did not contain at least two SNVs in that individual. (*) indicates significant icd-ASE (multiple testing adjusted p-vale < 0.05) in that individual. Y-axis, nominal GeneiASE p-value.

We applied GeneiASE on the pair of LPS treated and untreated white blood cell samples of each individual and identified eleven unique genes exhibiting significant icd-ASE (FDR ≤ 5%). We used pre-filtering (at least two variants present in dbSNP) and a filter based on the static ASE analysis (Methods). Next, we performed a meta-analysis across the eight individuals, in effect treating them as biological replicates, using the GeneiASE p-values from each individual as input (Methods). This analysis yielded 17 genes with significant cd-ASE (FDR ≤ 5%). Combining this set with the eleven genes identified in the single-individual analysis resulted in a total of 19 genes exhibiting condition-dependent ASE (0.2% of the 10,231 genes with heterozygous variants), Table 1 and Fig. 3 (more details in Supplementary Tables S5 and S6). Nine of these genes have previously been explicitly linked to inflammatory response (Supplementary Table S5).

We applied GeneiASE in static ASE mode to each of the 16 LPS treated or untreated white blood cell samples, including the pre-filtering (Methods). We found that 1.4% to 4.5% of the genes showed significant static ASE in the samples (FDR ≤ 5%), resulting in a total of 935 unique genes. Performing a meta-analysis across all samples with GeneiASE p-values as input yielded 1389 significant genes, Supplementary Tables S7 and S8. In all we detected static ASE in 13.6% of the 10,231 genes with heterozygous variants (Table 1).

### GeneiASE detection of cd-ASE was robust and consistent

We tested whether GeneiASE cd-ASE detection was robust with respect to artificially introduced mapping bias by perturbing the read counts of our LPS-treated or untreated samples. The perturbation was performed such that the reference allele read counts were increased while the alternative allele read counts were decreased (Supplementary Methods). In the individual cd-ASE analysis, 14 genes were detected from the perturbed data, including all eleven originally detected genes. The three additional genes were all borderline significant (adjusted p-value in the range 0.05–0.06) in the individual cd-ASE analysis of original (unperturbed) data, and two were detected in the original meta-cd-ASE analysis. We also performed the meta-analysis on the perturbed data. Combining the meta-cd-ASE genes with the set identified in the single-individual analysis resulted in a total of 22 genes exhibiting cd-ASE in the analysis of perturbed data. Four of these were new in the perturbed set, but borderline significant in the original analysis. In total, 95% (18/19) of the originally detected cd-ASE genes were detected also after the perturbation (Supplementary Table S9 and Supplementary Methods).

Next, we investigated whether the direction of GeneiASE cd-ASE effects was consistent between individuals (for the original, unperturbed data), *i.e.*, whether the sign of the log-odds-ratio was the same in all individuals that carried the variant. Among the 186 variants in the 19 cd-ASE genes, there were 51 variants (in 14 genes) that were shared by more than one individual. Forty-seven of the 51 variants (92.2%) had a cd-ASE direction that was consistent between all individuals in which the variant was present (Supplementary Table S6).

We concluded that the GeneiASE cd-ASE results were robust to artificially introduced reference-allele mapping bias, and that the consistency of cd-ASE direction across individuals was high.

### Experimental validation of ASE

**Figure 4:**
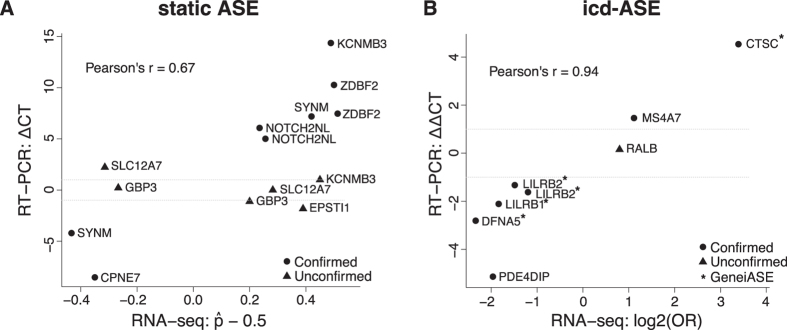
Correlation of ASE effect sizes from RNA-seq and real-time quantitative RT-PCR. (**A**) Static ASE. Y-axis, RT-PCR ASE effect size: ΔCT = ASE_RT-PCR_(ref,alt) = CT(ref) - CT(alt), *i.e*., difference in mean cycle threshold between the two alleles. X-axis, RNA-seq ASE effect size (Eq. 1, but without taking the absolute value). (**B**) icd-ASE. Y-axis, RT-PCR icd-ASE effect size: ΔΔCT = ΔASE_RT-PCR_(T-U) = ASE_RT-PCR_(T) – ASE_RT-PCR_(U). X-axis, RNA-seq ASE effect size ΔASE_RNA-seq_ (Eq. 2). Circles, confirmed by RT-PCR. Triangles, not confirmed. For icd-ASE: (*) indicates the five variants from the four genes detected as significant by GeneiASE.

**Figure 5:**
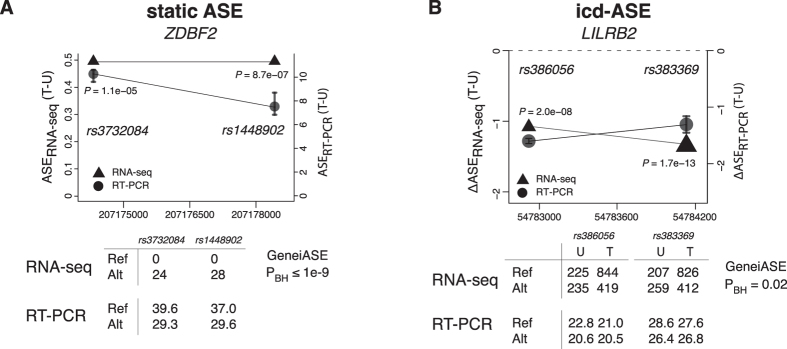
ASE analysis results for the genes *ZDBF2* and *LILRB2.* (**A**) Static ASE: *ZDBF2*. The two detected heterozygous variants in *ZDBF2*, *rs3732084* and *rs1448902*, showed complete static ASE in the RNA-seq data and a large difference in CT (cycle threshold) in the RT-PCR experiments. Upper panel: Left y-axis and triangles show the RNA-seq based static ASE effect size of the two variants (Eq. 1 in Methods); P-values are from the individual variant test. Right y-axis and circles show the RT-PCR based static ASE effect size (difference in CT). Error bars indicate standard error from the multiple RT-PCR experiments. Lower panel: Upper table shows RNA-seq read counts for the reference (Ref) and alternative (Alt) allele, with the resulting GeneiASE P-value, P_BH_; lower table shows RT-PCR CT values for ref and alt allele. All data are from the treated sample of individual i3 (3_T). (**B**) icd-ASE: *LILRB2*. The two detected heterozygous variants in *LILRB2*, *rs386056* and *rs383369*, showed icd-ASE in both RNA-seq data and RT-PCR experiments. Upper panel: Left y-axis and triangles show the RNA-seq based icd-ASE effect size of the two variants (Eq. 2 in Methods); P-values are from the individual variant test. Right y-axis and circles show the RT-PCR based icd-ASE effect size, ΔASE_RT-PCR_(T-U) = ASE_RT-PCR_(T) − ASE_RT-PCR_(U), where ASE_RT-PCR_ is the difference in mean CT values between the two alleles in either of the conditions T (treated with LPS) or U (untreated). Error bars indicate standard error from the multiple RT-PCR experiments. Lower panel: Upper table shows RNA-seq read counts for the Ref and Alt alleles and in the two conditions U and T, with the resulting GeneiASE P-value, P_BH_; lower table shows RT-PCR CT values for Ref and Alt alleles in the two conditions U and T. All data are from the untreated and treated samples of individual i6 (6_U and 6_T). P_BH_ indicates Benjamini-Hochberg corrected P.

Eight icd-ASE variants within 7 different genes were subjected to independent validation by real-time quantitative RT-PCR (Table 2 and Supplementary Table S10). We demanded the RT-PCR effect size, ΔASE_RT-PCR_(T-U), to be greater than 1, corresponding to at least a 2:1 ratio of alternate vs. reference (or vice versa) allele occurrence (Methods). RT-PCR confirmed changing ASE for seven variants corresponding to an observed FDR of 13% (Table 3). The overall correlation between RNA-seq and RT-PCR icd-ASE effect sizes was strong (Pearson’s r = 0.94), Fig. 4. Five of the variants were in four GeneiASE cd-ASE genes with more than one heterozygous variant: *CTSC*, *DFNA5*, *LILRB1*, and *LILRB2* (effect size correlation r = 0.997). Interpreting the validation results at the gene level, all four genes were confirmed to show icd-ASE (observed FDR = 0%) (Table 2, Table 3 and Supplementary Table S10). The variant that failed validation was in the gene *RALB*, and it had the lowest RNA-seq based effect size (and highest p-value from the Fisher’s exact test) of the eight tested. The RNA-seq and RT-PCR results for *LILRB2*, a receptor for class I MHC antigens^28^, are shown in Fig. 5.

Using the same experimental setup, 14 static ASE variants within 8 genes were subjected to validation (Table 2 and Supplementary Table S10). RT-PCR confirmed static ASE for eight variants, corresponding to an observed FDR of 43% (Table 3). The RNA-seq and RT-PCR static ASE effect sizes were correlated (Pearson’s r = 0.67), Fig. 4. Fourteen variants in eight of the 935 significant GeneiASE static ASE genes were put forward to validation by RT-PCR (Table 2). Two of the genes, *KCNMB3* and *ZDBF2*, exhibited complete ASE, i.e., only one allele showed expression, although identified as heterozygous in the SNP-array genotyping. Five of the eight genes were successfully validated to show static ASE, requiring at least one tested variant within the gene to be positively validated, and all other variants to exhibit ASE in the same direction. The results for *ZDBF2*, which is known from previous studies to exhibit ASE^29^, are shown in Fig. 5. Only one gene, *KCNMB3*, lacked the validation of both attempted variants. The observed FDR for static ASE at the gene level was 38% (Table 3). The overall static and icd-ASE variant validation success rate was 68% (15/22).

### Comparison of findings between ASE-methods

We compared GeneiASE to three recent methods developed to detect ASE in genes, including the model by Skelly^15^, MMSEQ^23^, and MBASED^22^. We tested the methods on the RNA-seq data presented in this study. The first two methods indicated that all genes with heterozygous variants showed ASE, an unrealistic result that lacks support in previous studies. Skelly’s method failed due to not supporting unphased data while MMSEQ failed due to inadequate phasing. For MBASED, we tested both static and condition-dependent ASE as indicated above, and found 2462 and 8 genes, respectively. MBASED exhibited extreme run-times for genes with many SNPs in two-sample mode, and those genes were excluded from the comparison (Supplementary Methods). The overlap with GeneiASE results was 812 static and, using GeneiASE’s non-optimal comparison mode, 7 cd-ASE genes (Supplementary Table S11).

### Haplotype information was not necessary to detect genes with ASE

We assessed the consistency between using and not using haplotype-information in the detection of genes with ASE, by employing available RNA-seq data from HapMap individual NA12878, along with its haplotypes which had previously been inferred from genome sequencing of the family trio^6^. GeneiASE, which does not use haplotype information, and MBASED, in its corresponding unphased version, were tested, and the results showed that 78% (46/59) and 80% (68/85) of the genes with ASE overlapped with MBASED phased ASE detection, respectively (Supplementary Tables S12 and S13). We also used the NA12878 data set to investigate the prevalence of ASE swapping between haplotypes within a gene, something that could indicate errors in phasing or ASE prediction, or the existence of differential haplo-isoform expression. For GeneiASE, 5.1% (3/59) of the genes exhibited ASE towards both of the haplotypes, at different variants, while for unphased MBASED, 10.6% (9/85) of genes exhibited varying direction of the ASE, while none for phased MBASED (Supplementary Table S12 and Supplementary Methods). We concluded that explicit or assumed phasing was not necessary for detection of ASE in genes, although phasing is needed in order to determine if observed ASE is due to more complex patterns of differentially expressed haplo-isoforms.

## Discussion

The main target of our interest was condition-dependent ASE, since it provides a potential connection between phenotype and genomic variants, and has to date only been scarcely studied^21^,^22^,^26^ and never in a controlled and reproducible setting. The method we proposed for assessing ASE in genes, GeneiASE, used unphased data and as such would be possible to apply on all existing RNA-seq data sets (with sufficient depth) without the need for additional experiments or haplotype estimation, thus instantly generating additional transcriptional details to published results.

In our study we applied GeneiASE on the gene level but transcript coordinates may also be used to define the regions to investigate. The most popular general transcript reconstruction and differential expression tool, the CuffLinks and CuffDiff suite^30^, does not handle allelic imbalances in expression. On the other hand, MMSEQ^23^ reconstructs haplo-isoforms (i.e., performs phasing), but only 9% of the variants in our study were phased with this method despite the relatively deep sequencing. The MMSEQ phasing relies on population inference, and could potentially be improved by incorporating additional samples from, e.g., HapMap. Phasing is necessary to elucidate which haplo-isoforms cause the observed ASE, but our and previous studies showed that genes with ASE can be detected without this information^22^. GeneiASE is similar to MBASED^22^ in certain aspects - e.g., both are applicable to detect static as well as changing ASE - but differs in other aspects, e.g., GeneiASE makes no phasing assumptions, treats samples symmetrically in cd-ASE mode, and exhibits reasonable run-times also for genes with many SNPs. We also suggested a meta-analysis approach to leverage the information from several individuals, i.e., biological replicates, in a coherent way.

We used GeneiASE detection of static ASE to assist detection of cd-ASE, allowing also non-significant static ASE to be informative for cd-ASE. We made this design choice since our cd-ASE definition allows for absence of significant static ASE in one or both of the samples and since the mapping biases associated with static ASE detection makes it a comparably unreliable approach, underscored by the observed FDR which, at 43% for static ASE, was much higher than that of icd-ASE (13%).

Our estimate of static ASE prevalence, 3.3–6.3%, is on par with previous studies of primary cells^19^,^21^, and is substantially lower than estimates based on cell lines (approximately 20% or more). Significant genomic differences between cell lines and tissue samples have been shown^31^,^32^, and the observations made by us and others suggest that these differences extend to ASE and that the prevalence of ASE as estimated from cell lines may not be directly applicable to primary cells.

We noted that our analyses detected relatively few cd-ASE cases. Technical reasons account for this to some extent, for instance small effect sizes and read depth dependence coupled with rather conservative detection approaches (FDR ≤ 5% for GeneiASE). We detected 19 significant cd-ASE genes whereof seven overlapped with the eight genes identified by MBASED. Furthermore, many of the cd-ASE genes have previously been implicated in inflammatory response, and cd-ASE was confirmed in all tested variants in the four genes subjected to validation. The consistency in the direction of the cd-ASE between individuals showcasing the same variant was also high. Collectively, this suggests that the condition-dependent ASE effect is real. Alternative splicing could, however, in rare instances cause false positive cd-ASE calls due to the mapping bias effect (see Supplementary Methods for a discussion). Most likely, there is also a set of cd-ASE genes without transcribed variants, which would escape detection in a sequencing-based method.

We complemented our RNA-seq data with genotype data in order to include variants with mono-allelic expression, i.e., where one allele is not transcribed at all, and to ensure adequate variant calling from the RNA-seq data (Supplementary Table S1). Sixteen out of the 22 variants subjected to validation were indicated as heterozygous by the SNP-array (the other 6 were absent from the SNP chip). The observed FDR for variants with SNP-array support was higher than the observed FDR for all variants (Table 3). Thus, genotype data seemed useful mostly to enable detection of mono-allelic ASE, but not for reducing the overall FDR.

This study catalogued and described the extent of static and condition-dependent ASE in primary white blood cell mRNA before and during inflammatory response. In the future, the challenge remains to assess the functional relevance of such expression variation, and to establish the agreement between measured ASE of mRNAs and proteins. Transcripts with ASE variants may be associated to regulatory regions and disease phenotypes through linking to GWA studies and eQTL analyses, while identification of regulatory variants can be done by functional genomics studies of transcription factor binding or epigenetic modifications^6^,^7^.

In summary, we demonstrated the existence of condition-dependent and static allele-specific expression in human primary white blood cells through a transcriptome-wide RNA-seq study coupled with directed real-time quantitative RT-PCR validation experiments. We presented RNA-seq based methods for ASE analysis that were well calibrated, phasing-independent, experimentally validated, freely available, and compatible with existing RNA-seq data obtained using standard protocols. Further, we observed that SNP-arrays were a useful complement to detect cases of complete ASE, and that haplotype information was not necessary to detect genes with ASE. We anticipate that studies of both static and condition-dependent allele-specific expression in biological systems will become an increasingly important part of the exploration of the transcriptional and regulatory landscape in the cell.

## Methods

### Ethics statement

The study and all its experimental protocols were approved by the Local Ethics Committee (Lokala etikprövningsnämnden) in Stockholm, Sweden (approval 2009/1374–32). All experiments and data analyses were carried out in accordance with this approval. All participants gave informed consent.

### Sample collection, genotyping, and RNA-sequencing

Four females and four males giving informed consent were recruited. Peripheral blood was extracted and white blood cell fractions separated to be subsequently treated with lipopolysaccharide (LPS) of Escherichia coli O55:B5. Genotyping was performed using Illumina Omni 2.5M SNP-arrays. Total mRNA was extracted from the samples, and library construction and sequencing (Illumina HiSeq2000, read length 2 × 100 bases) were performed according to the manufacturer’s protocols. RNA-seq variant calling was performed using SAMtools mpileup (including samtools view –q1, which discards all non-uniquely mapped reads). All variants in regions with overlapping features were discarded. Additional details are in Supplementary Methods.

### Allele-specific expression (ASE) analysis

The allele counts for the reference and alternative allele were extracted from individual-specific VCF files. Only variants present in dbSNP were retained for the ASE analysis. A minimum depth of 10 reads within a single individual was imposed since a variant needs to have sufficient depth to reach a useful power in the ASE analysis. All statistical analysis was done using R (R Core Team, 2014).

#### Static ASE

The allele-specificity of a heterozygous locus was assessed using the modified binomial test as described by Montgomery *et al.*^12^. To control for reference mapping bias, reads that overlapped any variant in the set of variants called as being heterozygous in the RNA-seq or the SNP-array data, and having a minimum depth of 10, were simulated as by Degner *et al.*^9^, generating an equal amount of reads for each of the two alleles at each heterozygous variant. After mapping the synthetic reads, the mapping ratio at each variant of the simulated reads was used as the new null hypothesis in a binomial test, rather than 0.5^12^, see also Fig. 1. We defined the effect size of static ASE for a variant, ASE_RNA-seq_, as the fraction of reads at the variant position mapping to the alternative allele, minus 0.5 and then taking the absolute value, Equation 1:

ASE_RNA-seq_ = | p − 0.5 | (Eq. 1), where p = c_alt_/(c_alt_ + c_ref_), and c is the read count and alt = alternative allele, ref = reference allele.

Thus, an equal number of reads aligning to each of the two alleles would result in ASE_RNA-seq_ = 0 (absence of ASE), while a variant at which all reads mapped to one allele would have ASE_RNA-seq_ = 0.5 (complete ASE). ASE_RNA-seq_(U) refers to the ASE in untreated state while ASE_RNA-seq_(T) refers to the ASE in the treated state.

#### Individual condition-dependent ASE (icd-ASE)

We defined the effect size of RNA-seq based condition-dependent ASE for a variant, ΔASE_RNA-seq_, as the log-odds ratio between the treated (T) and untreated (U) condition, Equation 2:

ΔASE_RNA-seq_ = log_2_(Odds-Ratio) = log_2_(odds(p(T))/odds(p(U))) (Eq. 2), where odds = p/(1−p), and p = c_alt_/(c_alt_ + c_ref_), where c is the read count and alt = alternative allele, ref = reference allele.

We assessed the statistical significance of icd-ASE (ΔASE_RNA-seq_) for a single variant by applying Fisher’s exact test on the allele read counts observed in the two conditions within a single individual, followed by Benjamini-Hochberg multiple testing correction. To focus our analysis on the set of SNVs where the power to detect icd-ASE was high, we required for each SNV a total read depth of >=100, summed over both conditions and both alleles.

#### GeneiASE, a gene-based test of static and individual condition-dependent ASE

A gene-based test was devised which combines the effect sizes from individual variants into a gene-wise measure.

We model the read counts observed from two alleles of a heterozygous variant by a beta-binomial distribution, which can be viewed as an overdispersed binomial distribution. The beta-binomial can be parameterized by two parameters, p, which reflects the mean, and ρ, which determines the overdispersion (ρ = 0 gives an ordinary binomial distribution). To obtain a null model for a single variant a beta-binomial (BB) was fitted to allele counts retrieved from DNA sequencing data (step 1), BB^0^ ~ BB(p^0^, ρ^0^). Once such a variant null model has been estimated, the method processes each gene independently to generate its p-value, by calculating a gene test statistic (Fig. 1 and Suppl. Fig. S1, step 2) and a null distribution specific for that gene (Suppl. Fig. S1, step 3). We denote the count of reads mapping to SNV j, at allele a, in gene i as c_ija_. In the case of static ASE there is a 2 × 1 table of such counts for each SNV, whereas in the case of condition-dependent ASE there is a 2 × 2 table where we denote the treatment status as t, and the corresponding counts as c_ijat_. First, if a table contains a zero, a pseudo-count of 1 is added to every element of the table, as to avoid numerical issues with infinity values (Suppl. Fig. S1, step 2.1). Next, a SNV test statistic is calculated by dividing the absolute value of the estimated effect size with the estimated standard error of the effect size (Suppl. Fig. S1, step 2.2). The absolute value is needed since phasing information is unavailable and we normalize with the standard error to adjust for differing sample-sizes. In the case of static ASE we use the log-odds of the estimated mean amount of ASE (c_ij1_/(c_ij1_ + c_ij2_)) as the effect, where 1 and 2 indicate the alternative and reference allele, respectively. We use Wilson’s method to estimate the standard error^33^. In the case of icd-ASE we use the log-odds ratio of the 2 × 2 table as the effect and 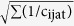 as the standard error estimate. Taking the log of the odds ratio provides a variance stabilization of the effect from individual variants. Once k SNV test statistics for a gene has been calculated they are combined into a gene test statistic by Stouffer’s method^34^ (Suppl. Fig. S1, step 2.3).

To obtain a null model specific for each gene, the variant null model BB^0^ is sampled N times for every SNV within a gene, using the observed read counts, c_ij_ (Suppl. Fig. S1, step 3.0). This generates N tables for each of the k SNVs within a gene, where N is the sample-size (default 10^5^). Pseudo-counts are then added and a SNV test statistic is calculated based on each table, in the same manner as in step 2.1 and 2.2, resulting in k SNV null distributions. The k variant null distributions are then combined to a gene null distribution by calculating a gene test statistic in the same manner as in step 2.3 for each of the N samples. Once such a gene null distribution has been obtained for gene i, the p-value can be calculated according to its definition (Suppl. Fig. S1 step 4). Since the null distribution of a gene depended on the number of variants within a gene, we generated one null distribution for each number of variants (k) up to k = 100 for which all genes with a greater number of variants were aggregated together.

#### Applying GeneiASE on our RNA-seq data

GeneiASE can be run on genes with ≥1 variant but in the main analysis of our RNA-seq data, we used pre-filtering such that GeneiASE was run on genes with ≥2 dbSNP variants. (GeneiASE results for genes with a single variant are in Supplementary Methods and Supplementary Tables S14 and S15). For cd-ASE, GeneiASE static ASE results were used to subset the set of genes available for cd-ASE analysis: only genes that exhibited a static ASE with nominal p-value ≤0.2 in at least one of the conditions were included in the cd-ASE analysis. Thus, we do not demand the presence of significant static ASE to detect cd-ASE, but allow weaker indications of static ASE to inform the cd-ASE test.

#### GeneiASE meta-analysis across individuals

To assess whether a gene exhibited ASE taking all eight individuals or 16 samples into account, we performed a meta-analysis by Fisher’s method, which is based on a multiplication of p-values rendering a chi-square statistic with two degrees of freedom under the null hypothesis: 
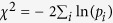
. We chose Fisher’s method since the genotypes often differ between individuals and its null hypothesis is that the null hypothesis is not rejected in any of the individual tests.

### Synthetic data generation

We generated synthetic RNA-seq data to assess (i) the FDR of the empirical data, and (ii) GeneiASE performance conditioned on varying properties of the data. Additional details are in Supplementary Methods.

### Real-time quantitative RT-PCR validation

Each sample was subjected to three independent RT-PCR validations, yielding technical triplicates of each cycle threshold (CT) value. The change of ASE between the two conditions in the RT-PCR experiments is defined as:


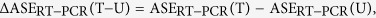


where ASE_RT-PCR_ is the difference in mean CT values between the two alleles for either condition (T or U). Additional details are in Supplementary Methods.

## Additional Information

**Accession codes:** The data sets supporting the results of this article are available in the following repositories: RNA-seq data are accessible at the NCBI Sequence Read Archive under accession number SRA062051 (http://www.ncbi.nlm.nih.gov/sra/?term=SRA062051). SNP-array data are accessible at ArrayExpress under accession number E-MTAB-1450 (https://www.ebi.ac.uk/arrayexpress/experiments/E-MTAB-1450/). Our software for detection of static and condition-dependent ASE, GeneiASE, along with instruction for its use, is available free of charge at http://geneiase.sourceforge.net.

**How to cite this article**: Edsgärd, D. *et al.* GeneiASE: Detection of condition-dependent and static allele-specific expression from RNA-seq data without haplotype information. *Sci. Rep.* **6**, 21134; doi: 10.1038/srep21134 (2016).

## Supplementary Material

Supplementary Information

Supplementary Table S2

Supplementary Table S5

Supplementary Table S6

Supplementary Table S7

Supplementary Table S8a

Supplementary Table S8b

Supplementary Table S10

Supplementary Table S13

Supplementary Table S14

Supplementary Table S15

## Figures and Tables

**Table 1:** Description of samples: sequencing, coverage, accessible loci, and loci with significant ASE.

Sample	Number of mapped high quality reads (Millions)	CCDS mean coverage	Loci with heterozygous dbSNP SNV and depth >10		Loci with significant(*) static ASE		Loci with significant(*) icd-ASE	
			SNVs	Genes	SNVs (%)	Genes (%)	SNVs	Genes
1_T	91	173	25749	4206	1240 (4.8)	117 (2.8)	40	2
1_U	60	114	23814	3906	785 (3.3)	82 (2.1)		
2_T	126	230	36680	4681	2096 (5.7)	184 (3.9)	55	2
2_U	75	142	25751	4070	1272 (4.9)	108 (2.7)		
3_T	99	185	28268	4392	1384 (4.9)	110 (2.5)	24	1
3_U	102	189	31163	4612	1698 (5.4)	124 (2.7)		
4_T	108	154	23069	3662	1152 (5.0)	87 (2.4)	36	2
4_U	130	184	31343	4436	1401 (4.5)	113 (2.5)		
6_T	137	189	32139	4408	1495 (4.7)	95 (2.2)	31	3
6_U	79	112	21777	3745	828 (3.8)	72 (1.9)		
7_T	177	282	24332	3965	1480 (6.1)	85 (2.1)	10	2
7_U	47	66	12478	2534	489 (3.9)	33 (1.3)		
8_T	267	358	55355	5888	3467 (6.3)	264 (4.5)	12	1
8_U	57	78	15968	2967	536 (3.4)	42 (1.4)		
9_T	63	110	22261	3556	838 (3.8)	62 (1.7)	22	1
9_U	84	154	27640	4266	1205 (4.4)	121 (2.8)		
*Median*	*95*	*164*	*25750*	*4138*	*1256*	*101.5*	*27.5*	*2*
*Total unique*			*150887*	*10231*	*13021*	935** [1389***]	*211*	11** [19***]

All gene counts and percentages refer to the set of genes with ≥2 SNPs in dbSNP. CCDS, Consensus Coding DNA Sequence; SNV, single nucleotide variant; icd-ASE, individual condition-dependent ASE; *N*_U and *N*_T refer to the untreated and LPS treated samples, respectively, of individual *N*. Samples 1, 2, 6, 9 are from female. Samples 3, 4, 7, 8 are from male. (*) Multiple testing corrected p-value < 0.05; (**) results obtained using GeneiASE. (***) results including GeneiASE meta-analysis.

**Table 2:** The 22 variants selected for RT-PCR validation.

	Gene	Chr	Pos	dbSNP id	SNV type	Ref	Alt	Sample or individual§	*icd-ASE*			*Static ASE*		
									ΔASE_RNA-seq_ p-value	ΔASE_RNA-seq_ amp	ΔASE_RT-PCR_ amp	ASE_RNA-seq_ p-value	ASE_RNA-seq_ amp	ASE_RT-PCR_ amp
***icd-ASE***	*PDE4DIP*	1	144873887	rs1699787	syn	C	T	9	7.9e-07	−1.88	−5.2#			
	*RALB*	2	121050989	rs1065518	3’UTR	T	A	1	1.4e-02	0.83	0.2			
	*DFNA5*	7	24758753	rs754555*	syn	C	T	4	3.5e-08	−2.32	−2.9#			
	*MS4A7*	11	60152563	rs950803	syn	T	A	3	1.3e-05	1.21	1.3#			
	*CTSC*	11	88045583	rs217086*	non-syn	A	G	2	1.9e-84	3.39	4.6#			
	*LILRB1*	19	55148487	rs8101605*	3’UTR	G	A	9	2.5e-12	−1.71	−2.1#			
	*LILRB2*	19	54782919	rs386056	non-syn	C	T	6	2.0e-08	−1.07	−1.6#			
	*LILRB2*	19	54784130	rs383369	non-syn	T	C	6	1.7e-13	−1.33	−1.3#			
***Static ASE***	*GBP3*	1	89474818	rs17433780*	non-syn	A	G	8_T				6.0e-101	−0.26	0.4
	*GBP3*	1	89479063	rs4656077*	non-syn	G	A	8_T				3.5e-47	0.20	−1.4
	*NOTCH2NL*	1	145282405	rs2596058	3’UTR	T	C	8_U				1.1e-04	0.24	6.2#
	*NOTCH2NL*	1	145282904	rs28713400	3’UTR	A	G	6_U				2.1e-05	0.26	5.1#
	*ZDBF2*	2	207174316	rs3732084*	syn	T	C	3_T				1.1e-05	0.50	10.3#
	*ZDBF2*	2	207178422	rs1448902*	3’UTR	G	A	3_T				8.7e-07	0.50	7.5#
	*KCNMB3*	3	178978604	rs7429685*	intronic	T	G	2_U				4.1e-41	0.49	14.4#
	*KCNMB3*	3	178978689	rs62410373*	intronic	A	G	2_U				1.9e-12	0.45	0.9
	*SLC12A7*	5	1057615	rs2241606*	syn	A	G	2_U				2.6e-11	0.29	−0.2
	*SLC12A7*	5	1081767	rs4526148*	non-syn	C	T	2_U				1.4e-09	−0.31	2.3
	*EPSTI1*	13	43542677	rs7324030*	intronic	C	A	3_T				3.8e-05	0.39	−1.8
	*SYNM*	15	99670518	rs1670227*	non-syn	T	C	7_T				2.7e-25	−0.43	−4.2#
	*SYNM*	15	99672722	rs2292288*	non-syn	A	G	7_T				7.1e-16	0.42	7.2#
	*CPNE7*	16	89663072	rs659974*	3’UTR	G	A	4_T				9.9e-03	−0.35	−8.5#

(#) Cases where the RT-PCR data validated the RNA-seq data (true positives). (*) SNV interrogated by SNP-array (heterozygous in the individual chosen for validation). (§) Sample chosen for validation and for which ASE amplitudes and p-value are shown. *N*_U and *N*_T refer to the untreated and LPS treated samples, respectively, of individual *N*. ASE_RNA-seq_ amplitude (amp) for static ASE and ΔASE_RNA-seq_ for icd-ASE are defined in Eq.1 and Eq 2, respectively (Methods). ASE_RT-PCR_ refers to the difference in cycle-threshold between the two alleles and ΔASE_RT-PCR_ = ASE_RT-PCR_(T) − ASE_RT-PCR_(U), where T and U corresponds to the treated and untreated condition. ASE_RNA-seq_ p-values: static ASE (modified binomial test), icd-ASE (Fisher’s exact test). P-values were Benjamini-Hochberg multiple testing corrected. Chr, chromosome; Pos, SNV coordinate on chromosome; Ref, reference allele; Alt, alternative allele; icd-ASE, individual condition-dependent ASE; SNV, single nucleotide variant; syn, synonymous exonic; non-syn, non-synonymous exonic; UTR, un-translated region.

**Table 3:** False discovery rates for ASE detection.

ASE type	Detection method	Observed FDR
icd-ASE, SNV	Fisher’s exact test	13% (1/8)
icd-ASE, gene	GeneiASE	0% (0/4)
Static ASE, SNV	Modified binomial	43% (6/14)
Static ASE, gene	GeneiASE	50% (4/8)
All variants		32% (7/22)
All variants with SNP-array support		38% (6/16)

FDR, false discovery rate; SNV, single nucleotide variant; icd-ASE, individual condition-dependent ASE. The Observed FDR column represents false positives divided with the total number of variants subjected to validation within the category (actual numbers in parenthesis).
