# Supplementary material for: GeneiASE: Detection of condition-dependent and static allele-specific expression from RNA-seq data without haplotype information
Source: Sci Rep. 2016 Feb 18;6:21134. doi: 10.1038/srep21134 (PMC4758070; doi:10.1038/srep21134)
Supplement: Supplementary Information [file srep21134-s1.pdf]

Edsgärd et al., 2015

Supplementary Information: Supplementary Figures, Supplementary Tables, Supplementary Methods, and Supplementary References.

GeneiASE: Detection of condition-dependent and static allele-specific expression from RNA-seq data without haplotype information

Daniel Edsgärd[1,§], Maria Jesus Iglesias[2, 3,§], Sarah-Jayne Reilly[2,§], Anders Hamsten[2], Per Tornvall[4], Jacob Odeberg[2, 3, 5,*], and Olof Emanuelsson[1,*]

[1] KTH Royal Institute of Technology, Science for Life Laboratory, School of Biotechnology, Division of Gene Technology, SE-171 65, Solna, Sweden
[2] Atherosclerosis Research Unit, Department of Medicine Solna, Karolinska Institutet; Center for Molecular Medicine; and Department of Cardiology, Karolinska University Hospital, Stockholm, Sweden.
[3] KTH Royal Institute of Technology, Science for Life Laboratory, School of Biotechnology, Division of Proteomics, SE-171 65, Solna, Sweden
[4] Department of Clinical Science and Education, Södersjukhuset, Karolinska Institutet, Stockholm, Sweden
[5] Department of Medicine, Centre for Hematology, Karolinska University Hospital and Karolinska Institutet, Solna, Sweden

* To whom correspondence should be addressed.

Corresponding author: Olof Emanuelsson; Tel: +46-8-52481458; Fax: +46-8-52481425; Email: olofem@kth.se

Correspondence may also be addressed to: Jacob Odeberg; Tel: +46-708-2087571; Email: jacob1@kth.se

(§) Joint first authors.

SUPPLEMENTARY FIGURES

1. Estimate beta-binomial (BB) model parameters, p^0, ρ^0, from DNA allelic counts (c_a^0).

$$c_a^0 \sim BB(p^0, \rho^0, c^0) = BB(\alpha, \beta, c^0), where\, p^0 = \frac{\alpha}{\alpha+\beta}, \rho^0 = \frac{1}{1+\alpha+\beta}$$

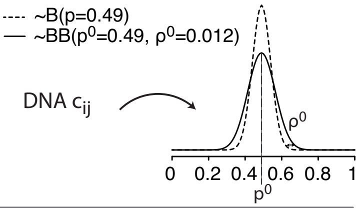

2. Calculate gene test-statistics, g_i, for a gene i, with k SNVs (j).
The input is:
static-ASE: k 2x1 tables, containing counts for each allele a: c_{ija}
icd-ASE: k 2x2 tables, containing counts for each allele in each treatment t: c_{ijat}

2.1. Add pseudo-count to tables containing any zeros

$$c_{ijat} = c_{ijat} + I_j, I_j = \begin{cases} 0, & \text{if } c_{ijat} \neq 0\, \forall c_{ijat} \in c_{ij} \\ 1, & \text{otherwise} \end{cases}$$

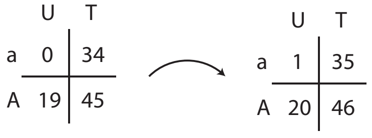

2.2. Calculate SNV test statistics

$$s_{ij} = \frac{|eff|}{SE(eff)}, eff = \begin{cases} log(odds(\hat{p})), & \text{if static-ASE} \\ log(OR(\hat{p}|_{t=1}, \hat{p}|_{t=0})), & \text{if icd-ASE} \end{cases}$$

$$\hat{p} = \frac{c_{ij}|_{a=alt}}{c_{ij}|_{a=alt} + c_{ij}|_{a=ref}}$$

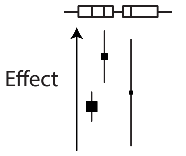

2.3. Calculate gene test statistics by pooling SNV effects according to Stouffer's method

$$g_i = \frac{\sum_{j=1}^{k} s_{ij}}{\sqrt{k}}$$

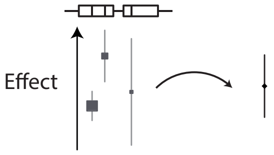

3. Generate null distribution, g_i^0, for the gene i
The input is:
p^0, ρ^0, c_{ij}, N, where N = number of samples drawn (default = 10^5)

3.0. Sample allelic counts from parametric model with parameters estimated from DNA (step 1 above). The sampling will output: N x k tables.
static-ASE: $c_{ija} \sim BB(p^0, \rho^0, c_{ij}^0 = c_{ij})$

icd-ASE: $\begin{cases} c_{ija}|_{t=0} \sim BB(p^0, \rho^0, c_{ij}^0 = c_{ij}|_{t=0}) \\ c_{ija}|_{t=1} \sim BB(p^0, \rho^0, c_{ij}^0 = c_{ij}|_{t=1}) \end{cases}$

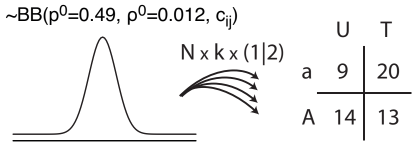

3.1. as in 2.1

3.2. as in 2.2, generating k x s_{ij} distributions, each with N values

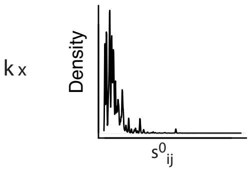

3.3. as in 2.3, generating a g_i^0 distribution of N values

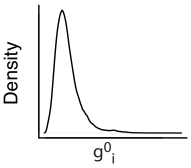

4. Calculate p-value for gene i, given its null distribution g_i^0

$$pval = \frac{\sum_{l=1}^{N} I_l}{N}, I_l = \begin{cases} 1, & \text{if } g_{il}^0 \geq g_i. \\ 0, & \text{otherwise.} \end{cases}$$

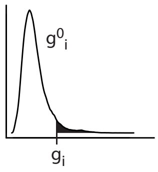

Figure S1

A detailed description of each step is provided in Methods. 1. A null model for allelic counts from a single variant is estimated from DNA data. 2. A gene-test statistic is

calculated for each gene by pooling the SNV test statistic belonging to the gene. 3. A null model specific for each gene is calculated according to the characteristics of the gene (number of SNVs and read depth of each SNV), by sampling from the SNV null model obtained in the first step. 4. Given the observed gene test statistic from step 2 and the null model from step 3, a p-value is obtained.

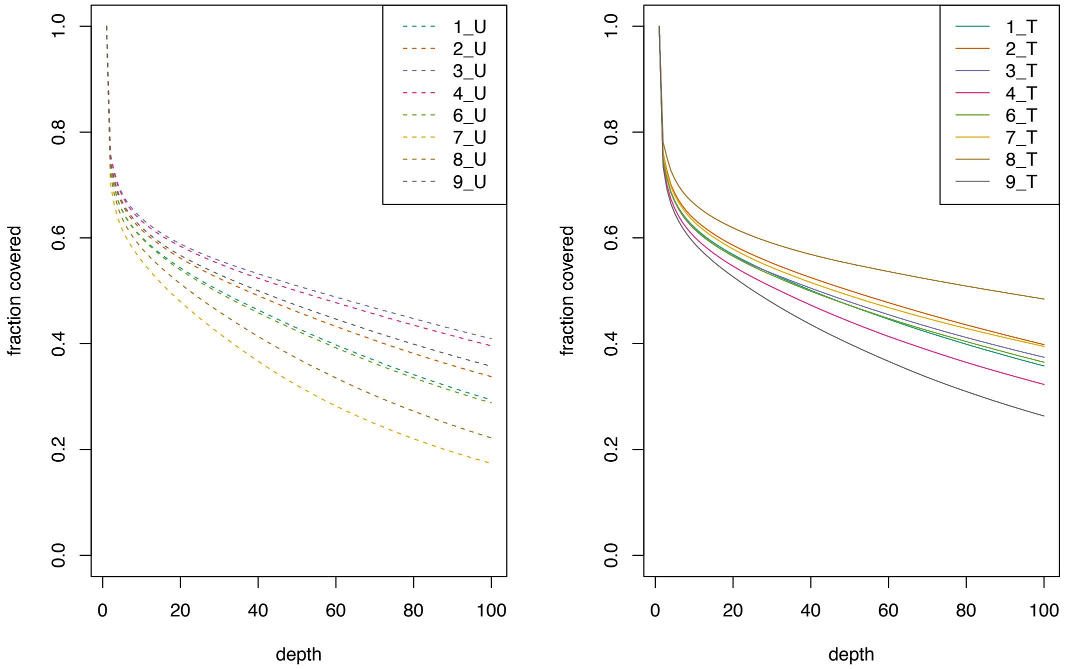

Figure S2. Fraction of CCDS (consensus coding DNA sequence) coverage for untreated samples (left panel) and LPS treated samples (right panel) at depths 1-100. Y-axis, fraction of CCDS covered. X-axis, sequencing depth (as present after mapping of reads).

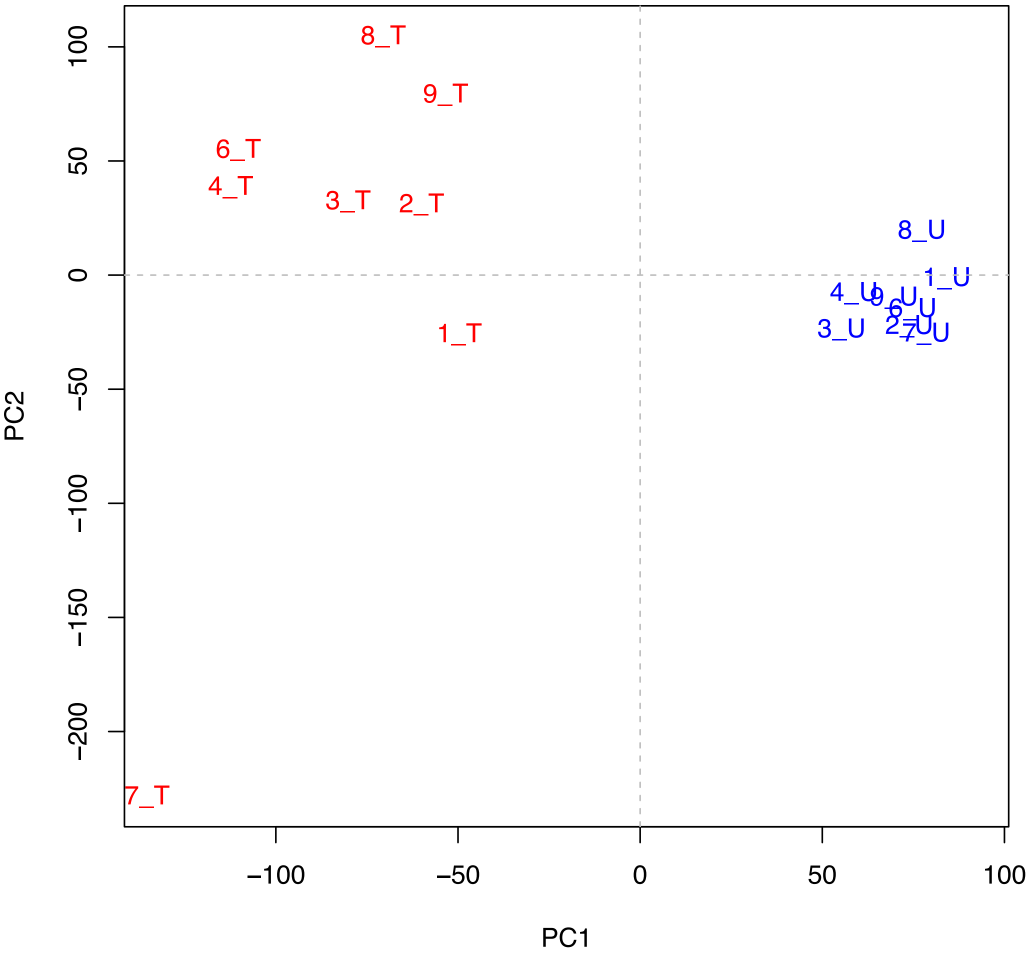

Figure S3. Principal component analysis of gene expression levels for the untreated (blue) and LPS treated (red) samples. We plot the first two principal components, PC1 and PC2.

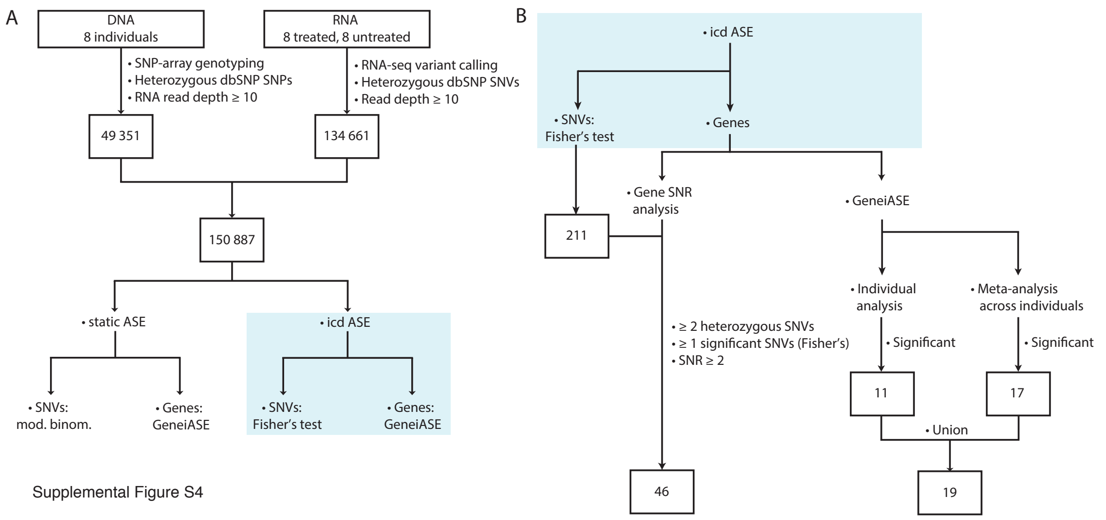

Figure S4. ASE analysis flowchart
(A) Variation calling pipeline leading to static ASE analysis at the lower left and (highlighted) individual condition-dependent ASE (icd-ASE) at the lower right. (B) icd-ASE analysis. SNV, single nucleotide variant; DE, differential expression; SNR, signal-to-noise ratio, SNR = mean/standard deviation, calculated for each gene based on icd-ASE effect sizes for the SNVs in the gene. The numbers of accessible variants and the numbers of resulting ASE cases are indicated in the Figure.

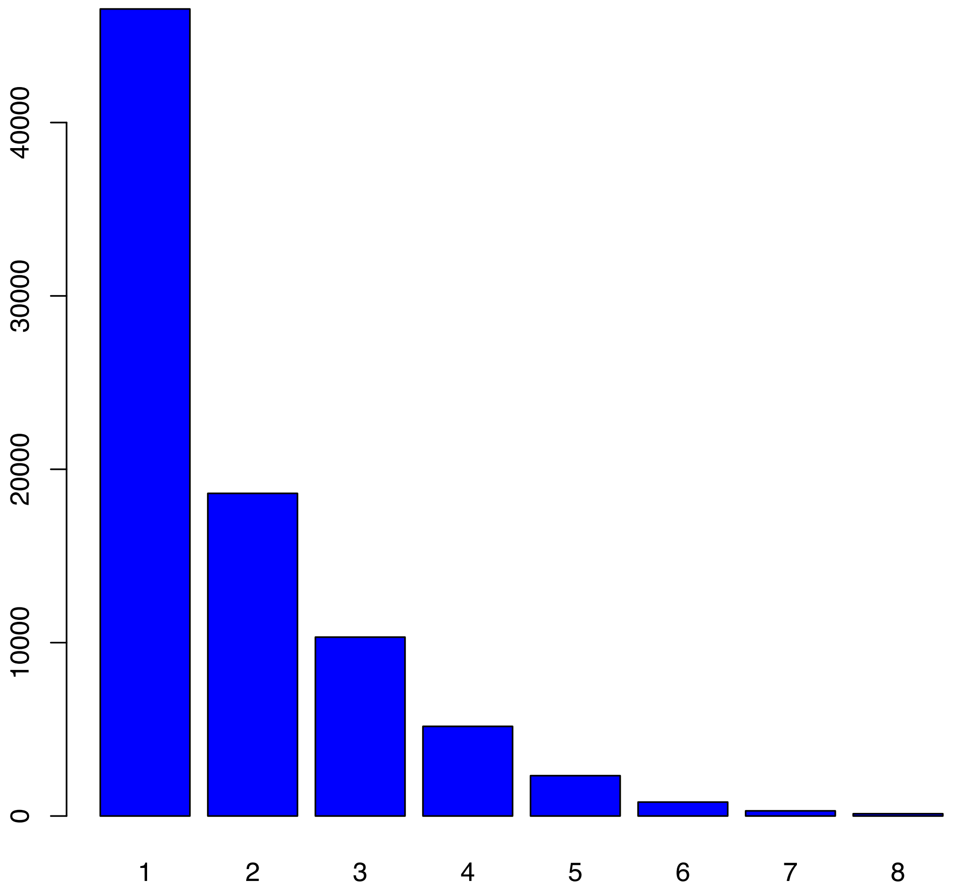

Figure S5. Minor allele frequency spectrum. Y-axis: number of SNVs; x-axis: the number of individuals where a SNV was observed.

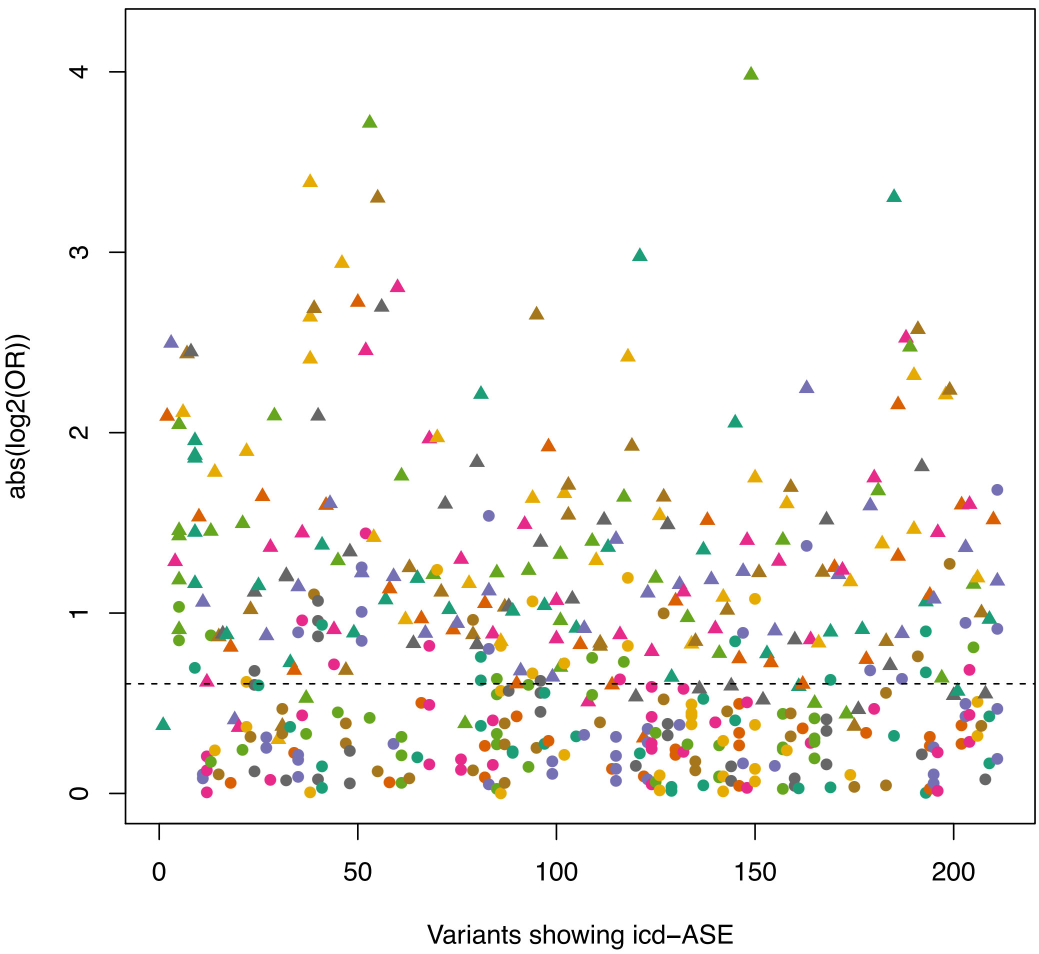

Figure S6. Sustained effect size across individuals for the 211 statistically significant icd-ASE variants. Y-axis, the absolute value of the difference in ASE between LPS-treated (T) and untreated (U) samples in an individual. X-axis, enumeration of the 211 variants and for each variant there are 8 values (corresponding to the 8 individuals used in this study). The dashed line corresponds to the 90th percentile of the observed amplitude of ASE changes among the 211 variants. 51 variants (24%) were above the threshold in at least one individual, and many in >1 individual (i.e., more than one dot/triangle above the dashed line). Color distinguishes SNVs and triangle indicates if a SNV had significant icd-ASE ($P \leq 0.05$, Benjamin-Hochberg corrected).

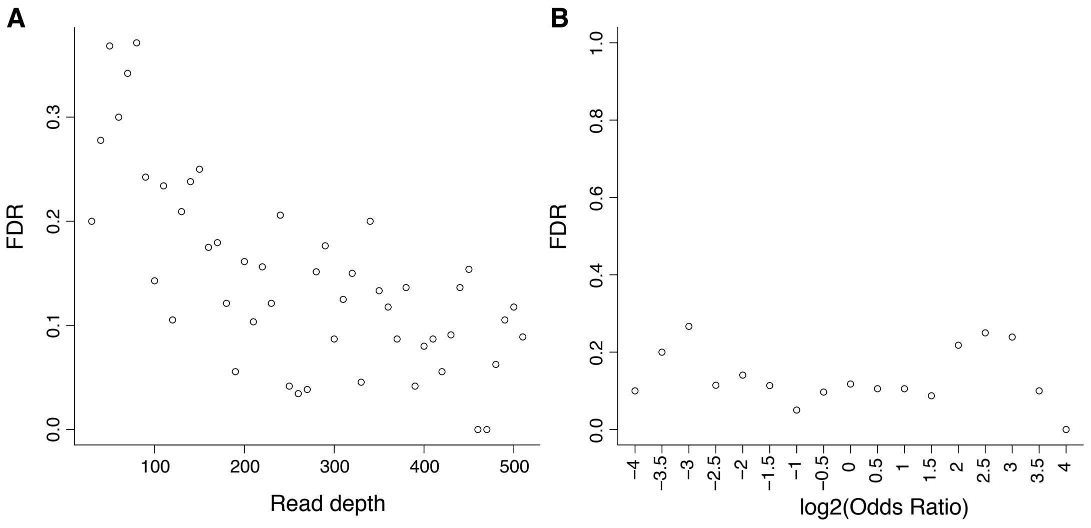

Figure S7. False discovery rate (FDR) of variants showing significant individual condition dependent ASE (icd-ASE), against read depth (A) and against effect size (log2 of the odds ratio) (B).

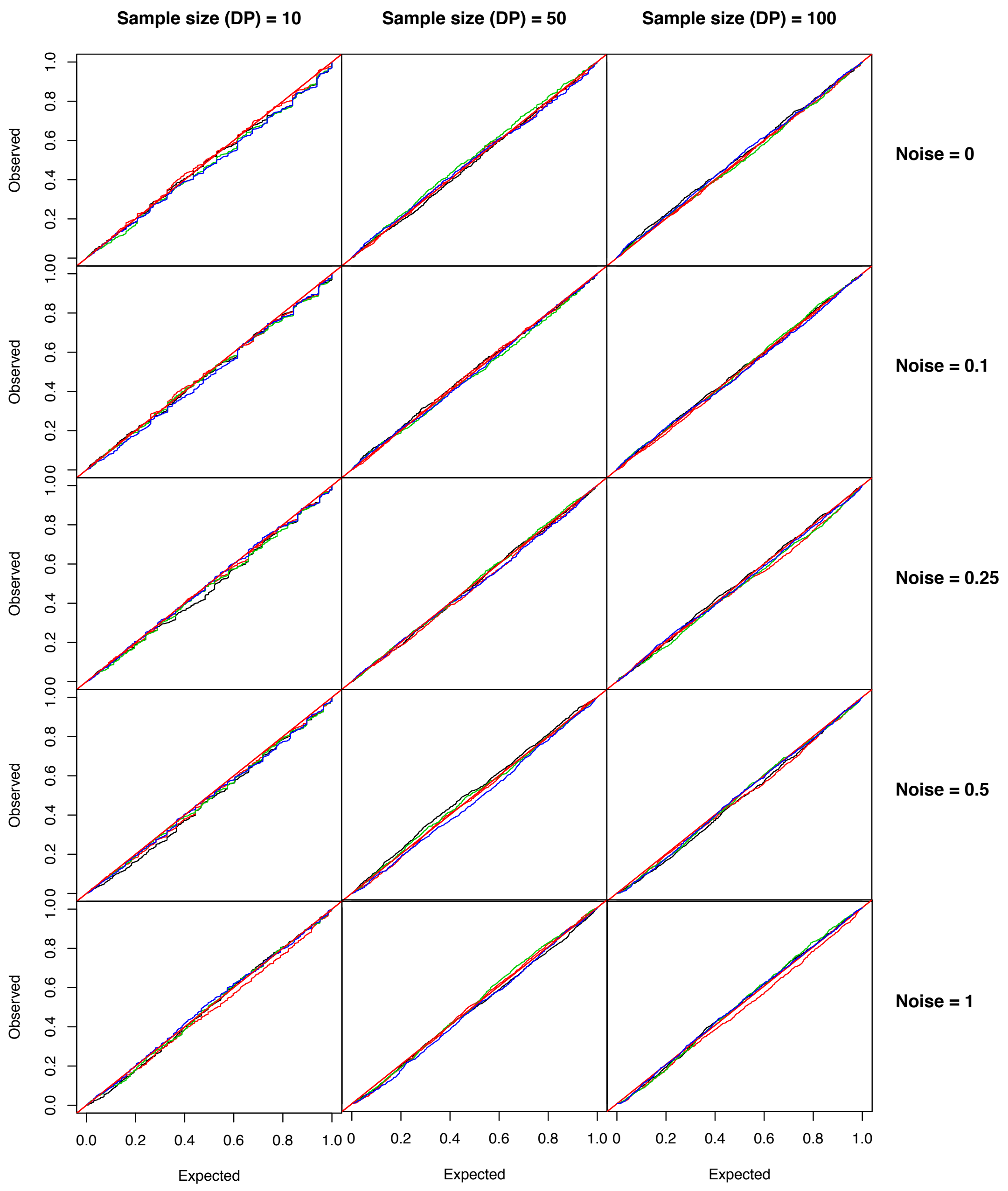

Figure S8. Calibration of static-ASE GeneiASE. The p-value distributions from GeneiASE are uniform under the null hypothesis of no static ASE, implying that observed FDRs reflect expected FDRs. Colors indicates simulation replicates. DP: read depth

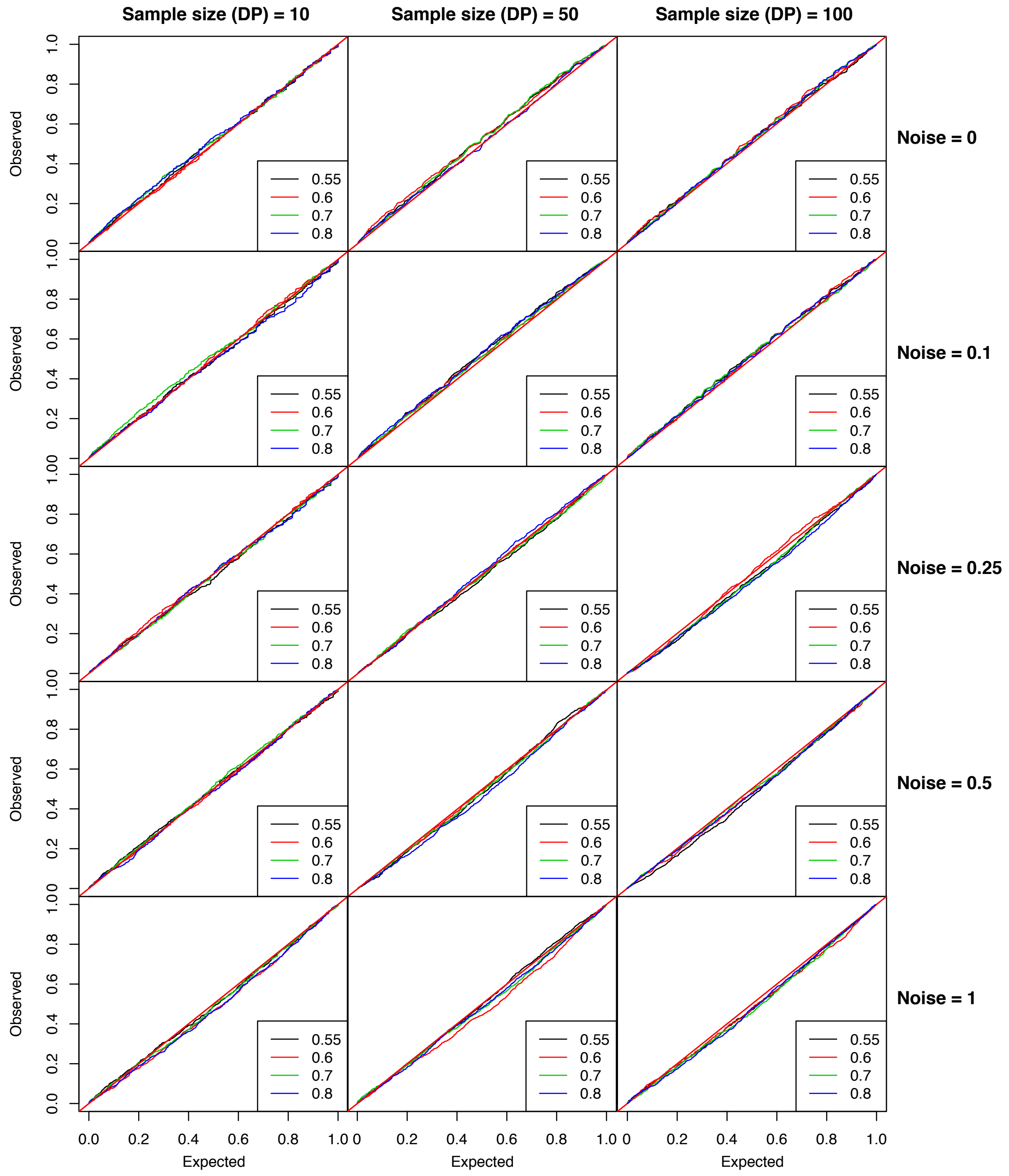

Figure S9. Calibration of icd-ASE GeneiASE. The p-value distributions from GeneiASE are uniform under the null hypothesis of no icd-ASE, implying that observed FDRs reflect expected FDRs. Colors indicate varying degrees of ASE (but which was identical for the untreated and treated condition), see inset in each panel. DP: read depth.

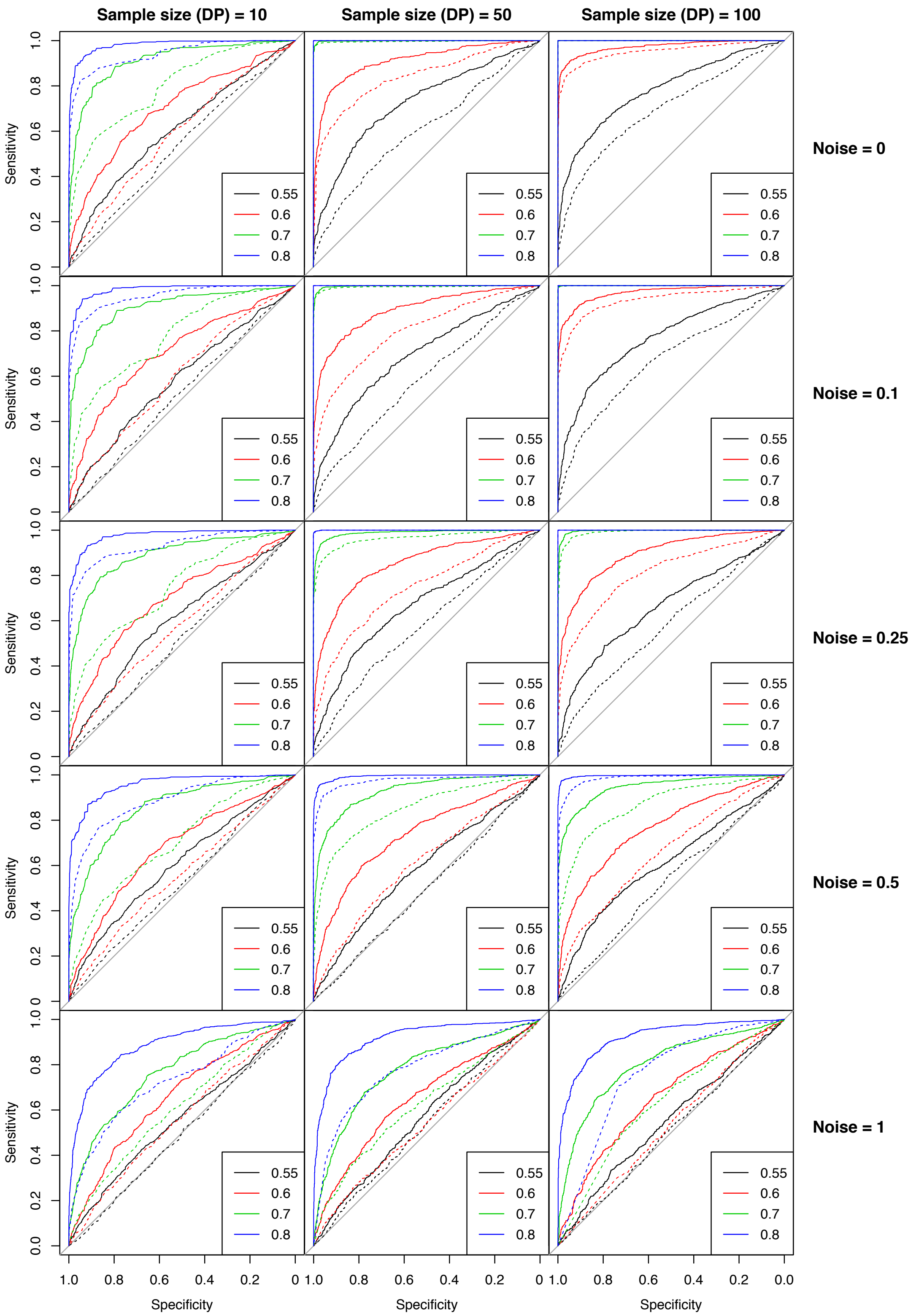

Figure S10. ROC-curves for static ASE show that GeneiASE consistently outperforms a simpler approach, under varying read depth, effect size and noise level. Dashed line indicates a simpler approach using meta-analysis of SNV test statistic of a gene, where a modified binomial test was used to calculate the SNV statistic (Methods). Color indicates the level of static ASE (see inset).

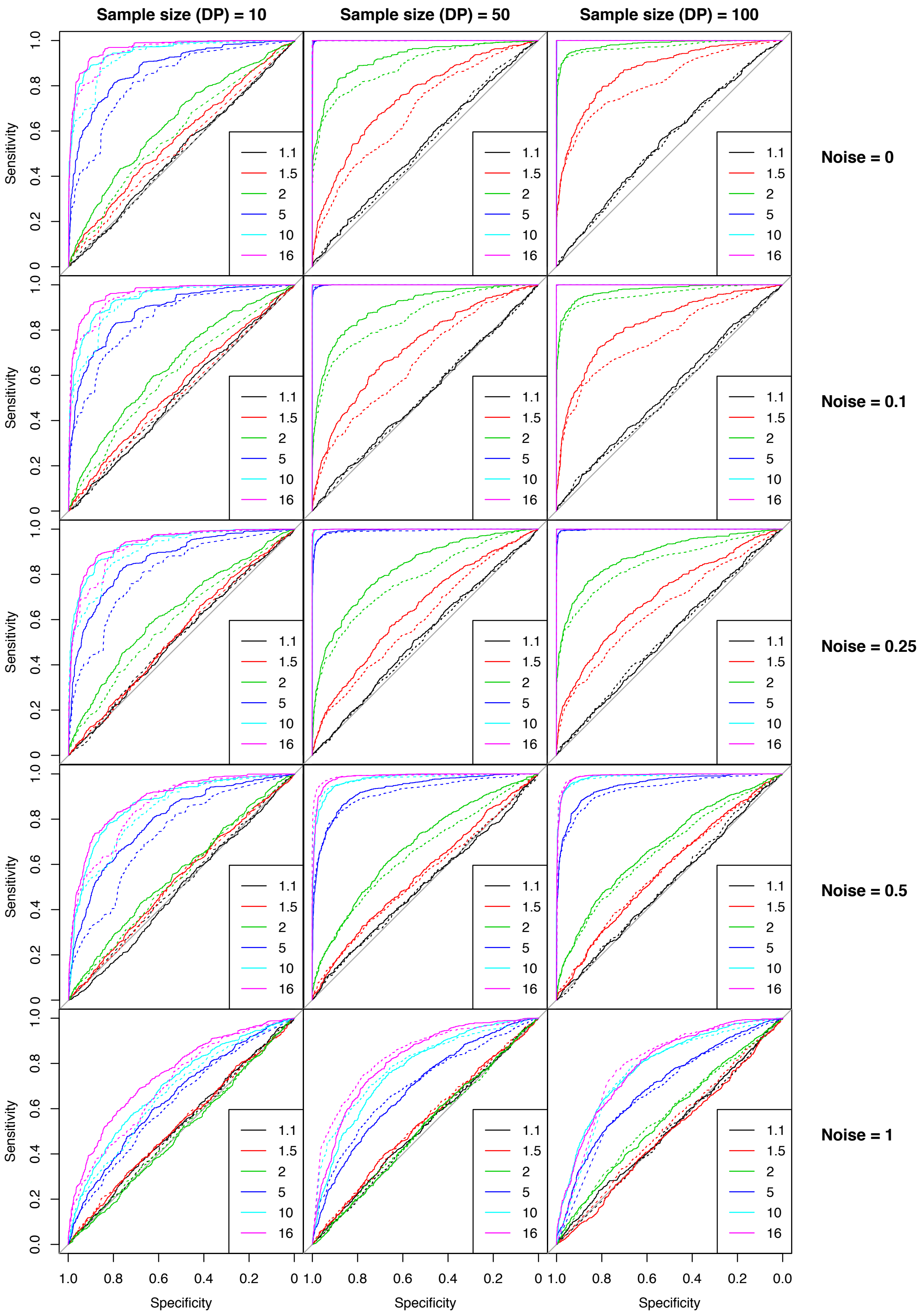

Figure S11. ROC-curves for icd-ASE show that GeneiASE consistently outperforms a simpler approach, under varying read depth, effect size and noise level. Dashed line indicates a simpler approach using meta-analysis of SNV test statistic of a gene, where Fisher's test was used to calculate the SNV statistic (Methods). Color indicates the level of icd-ASE (odds-ratio), see inset in each panel. DP: read depth

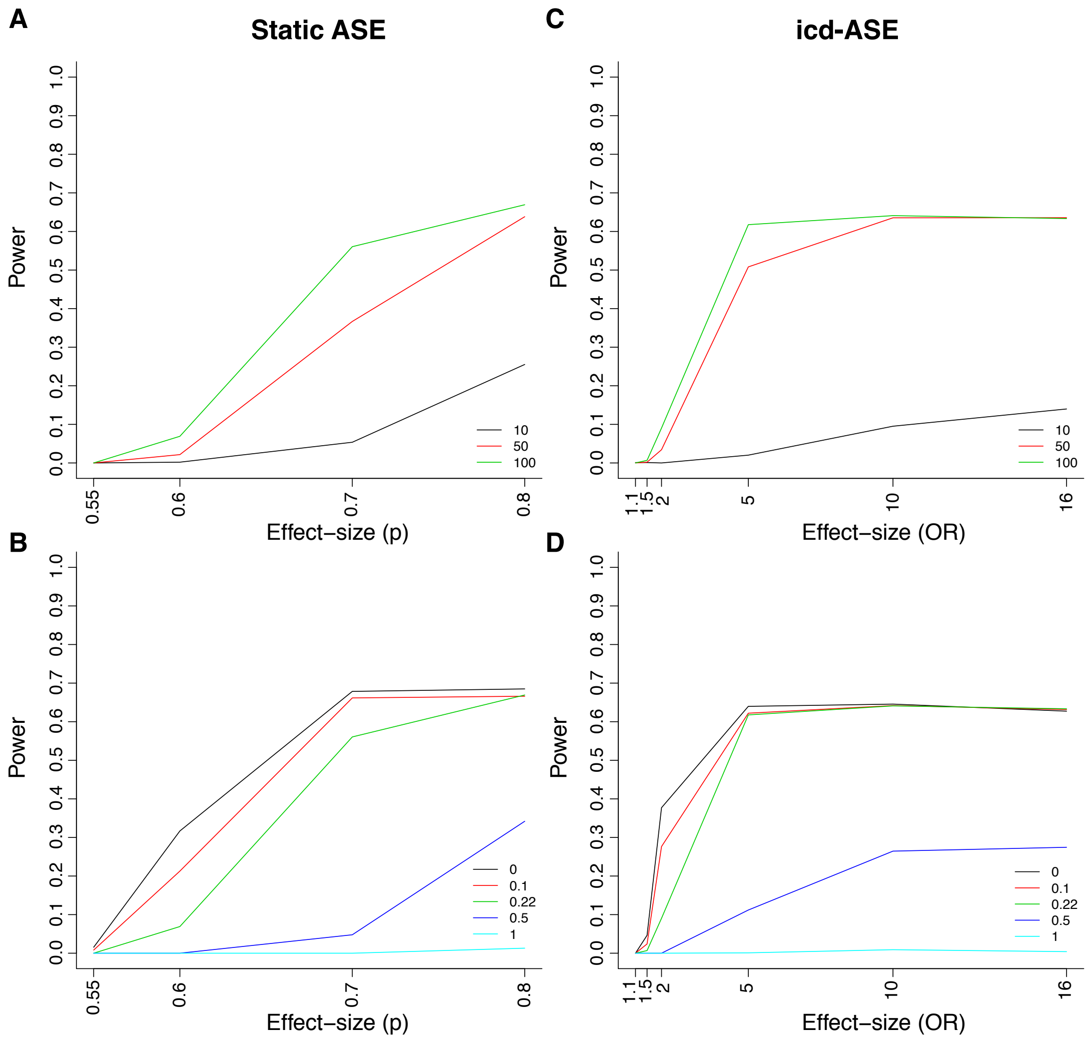

Figure S12. Power analysis of GeneiASE analysis, with respect to effect size. A-B show results for static ASE and C-D for icd-ASE analysis. Color indicates read depth (A, C), where noise was set to 0.22 as estimated from empirical data, or noise level (B, D), where read depth was set to 100, see inset.

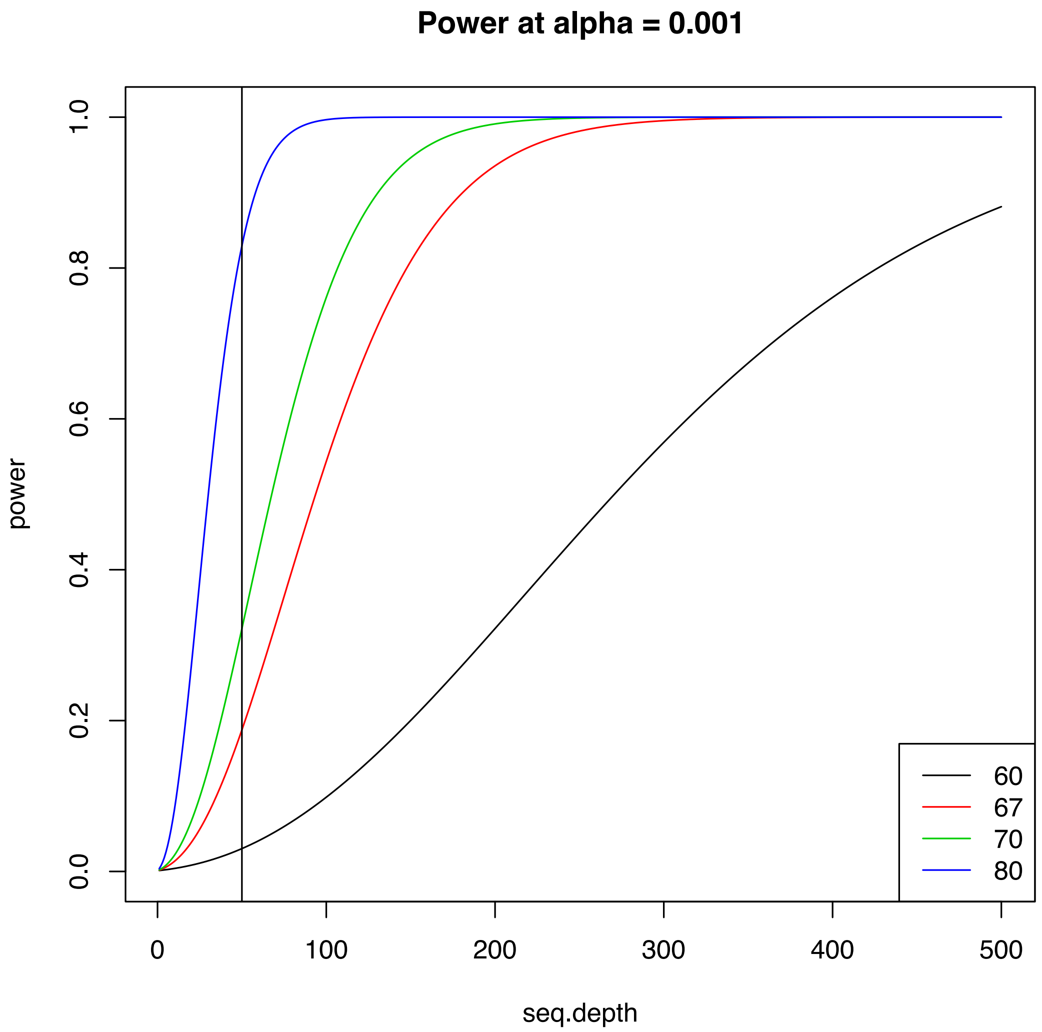

Figure S13. Sequencing depth power analysis. Y-axis indicates sensitivity at significance level alpha = 0.001. X-axis: read sequencing depth. At read depth 50 (leftmost vertical line) the sensitivity is above 80% for variants showing an allelic ratio of 80:20 (blue line). Black, red, green, and blue lines represent allelic ratio of 60:40, 67:33, 70:30, and 80:20, respectively (see inset).

SUPPLEMENTARY TABLES

Table S1. Concordance between heterozygous SNV calls from RNA-seq and SNP-array (Illumina Omni 2.5M). Called SNVs had a read depth of at least 10. Het, heterozygous.

Sample	RNA-seq het.SNVS	Het.SNVs (RNA-seq ∩ SNP-array)	Concordant het.SNVs ⊂ (RNA-seq ∩ SNP-array)	Disconcordant het.SNVs ⊂ (RNA-seq ∩ SNP-array)	Percentage het. SNVs (RNA-seq ∩ SNP-array) / RNA-seq	Percentage concordant het.SNVs ⊂ (RNA-seq ∩ SNP-array)
S1_LPS	20499	7519	6445	1074	36.7%	85.7%
S1_U	18667	6806	5830	976	36.5%	85.7%
S2_LPS	31257	10090	8885	1205	32.3%	88.1%
S2_U	20406	7314	6384	930	35.8%	87.3%
S3_LPS	22960	7815	6862	953	34.0%	87.8%
S3_U	25941	8743	7695	1048	33.7%	88.0%
S4_LPS	17868	6426	5610	816	36.0%	87.3%
S4_U	26052	8424	7383	1041	32.3%	87.6%
S6_LPS	26853	8617	7503	1114	32.1%	87.1%
S6_U	16606	6268	5389	879	37.7%	86.0%
S7_LPS	19615	7128	6233	895	36.3%	87.4%
S7_U	8205	3810	3259	551	46.4%	85.5%
S8_LPS	49629	14325	12860	1465	28.9%	89.8%
S8_U	11091	4688	4153	535	42.3%	88.6%
S9_LPS	17068	6134	5280	854	35.9%	86.1%
S9_U	22309	7598	6572	1026	34.1%	86.5%

Table S2. 211 variants showing significant icd-ASE (individual condition-dependent ASE). Ref, reference allele; Alt, alternative allele; BH, Benjamin-Hochberg multiple testing correction; ΔASE(T-U) ASE treated sample – ASE untreated sample.

See separate file Table_S2.Edsgard_et_al.2015.csv. (An .xlsx or .xls version is available upon request from the authors).

Table S3. **Signal-to-noise ratio (SNR) of 68 genes with at least two variants of which at least one showed significant icd-ASE (individual condition-dependent ASE).** The SNR (mean/standard dev.) was calculated using the icd-ASE effects of the variants within a gene.

Gene	# of individuals	Individual with max SNR	# of het.SNVs in gene	SNR
CECR1	8	1	3	5.0
PDE4DIP	8	2	2	4.5
PARP4	7	9	2	24.2
LILRB3	7	4	2	3.0
NUP210	7	3	6	2.5
MUS81	6	3	4	4.8
HTT	6	1	3	2.8

PSME4	6	3	4	2.7
PLXND1	6	4	7	2.3
GAA	6	8	4	2.2
DDX58	6	6	3	1.7
GLRX	5	2	2	6.2
CHSY1	5	1	2	4.5
SLC12A7	5	6	2	33.5
HECTD1	5	2	4	2.8
MED16	5	8	4	2.4
CD93	5	8	3	2.1
SMCO4	5	8	2	10.5
MEFV	5	7	5	1.7
DFNA5	4	2	2	70.9
MYOF	4	4	3	8.0
CXCL16	4	1	2	7.6
SLFN5	4	4	3	6.3
FAM208A	4	3	2	5.5
GBP3	4	2	2	5.0
WDR11	4	4	6	2.9
CNDP2	4	9	2	2.7
FCAR	4	9	2	13.8
TLR1	4	2	2	13.0
ABCC1	4	4	5	1.5
NPC1	3	3	2	6.8
LILRA1	3	6	2	5.0
CYFIP2	3	8	2	4.2
ODF2L	3	3	8	2.5
SP140L	3	6	4	2.1
BLMH	3	8	2	1.9
HLA-C	3	2	4	1.9
ARRB2	3	3	2	1.5
SULF2	2	4	2	8.9
LILRB2	2	9	2	7.0
SIGLEC5	2	8	2	6.7
TMEM176A	2	3	2	4.4
IL7R	2	6	3	4.2
HLA-B	2	4	2	3.9
MS4A7	2	2	3	3.8
P2RX7	2	3	3	3.8
LILRB4	2	2	2	2.9
PIM1	2	6	2	2.5
PGD	2	7	2	1.8
ELMO1	2	2	2	1.7
SAMSN1	2	2	8	1.6
KIAA1429	2	2	2	1.6
CSF1R	2	2	2	1.5

ITGB2	2	6	2	11.1
CD82	1	3	3	9.7
CD101	1	2	2	8.0
GPNMB	1	8	2	29.1
ASAH1	1	6	2	1.8
EIF2A	1	1	2	1.6
TMEM176B	1	1	3	1.5
FCGR3A	1	2	2	1.1
CUX1	1	2	2	1.1
TARP	1	9	2	0.9
SULT1A1	1	2	2	0.9
TBC1D10A	1	7	2	0.9
TPR	1	3	2	0.9
CSGALNACT1	1	3	2	0.8
BSG	1	8	2	0.8

Table S4. **Desired properties of methods used to detect cd-ASE in genes.** We identified six properties, P1-P6, that a well-powered model should incorporate to identify genes with condition-dependent allele-specific expression (cd-ASE) from RNA-seq data for individuals where the diploid genome is unavailable (unphased data). (P1) Paired model, since the data is from the same individual under the different treatment conditions. (P2) Binomial model, rather than a Poisson, since the marginal sum of the counts from two alleles is fixed. (P3) Random effect model, since the effect may vary along a gene (for different variants), due to, e.g., technical noise. (P4) Variance stabilization of effect sizes for condition-dependent ASE. (P5) Estimation of the null model from DNA. (P6) Undirected effect, i.e., independence of the effect directionality between variants, since data is unphased. MH, Mantel-Haenszel method [1]. (DL), DerSimonian-Laird [2]. LS, Liptak-Stouffer [3]. Skelly, (Skelly et al. 2011). MMSEQ, (Turro et al. 2011). Pham, (Pham and Jimenez 2012). MBASED, (Mayba et al. 2014). na, not applicable.

	**Desired property**	**GeneiASE**	**MH**	**DL**	**Fisher's test + LS**	**Skelly**	**MMSEQ**	**Pham**	**MBASED**
P1	Paired	x	x	x	x		x	x	x
P2	Binomial	x	x	x	x	x	na		x
P3	Random effect	x		x		x	x	x	x
P4	Variance stabilization	x				x	x		
P5	DNA null	x				x	na		
P6	Undirected effect	x			x				(*)

(*) MBASED performs pseudo-phasing on non-phased data using one of the two contrasted samples, causing the results to depend on the sample chosen for phasing

Table S5. All 19 genes with significant icd-ASE according to GeneiASE (including meta-analysis). GeneiASE results are shown for each individual. Genes are ordered according to their meta-analysis p-values.

See separate file Table_S5.Edsgard_et_al.2015.xls.

Table S6. All variants in the 19 significant icd-ASE genes detected by GeneiASE (including meta-analysis). Chromosomal location, dbSNP id, reference/observed allele, SNP location relative to gene annotation, variant type (synonymous/nonsynonymous), RNA-seq read depths and corresponding p-values for all variants in all individuals for the 19 genes with significant icd-ASE according to GeneiASE (including meta-analysis). There are in total 186 variants, whereof 51 (in 14 genes) were shared by more than one individual (see column n.individuals).

See separate file Table_S6.Edsgard_et_al.2015.xls.

Table S7. All 1389 genes with significant static ASE according to GeneiASE (including meta-analysis). GeneiASE results are shown for each individual. Genes are ordered according to their meta-analysis p-values.

See separate file Table_S7.Edsgard_et_al.2015.xls.

Table S8. All variants in the 1389 significant static ASE genes detected by GeneiASE (including meta-analysis). Chromosomal location, dbSNP id, reference/observed allele, SNP location relative to gene annotation, variant type (synonymous/nonsynonymous), and RNA-seq read depths and corresponding p-values for all variants in all individuals for the 1389 genes with significant static ASE according to GeneiASE (including meta-analysis).

See separate files Table_S8a.Edsgard_et_al.2015.csv and Table_S8b.Edsgard_et_al.2015.csv. (An .xlsx or .xls version is available upon request from the authors).

Table S9. Genes with different cd-ASE calls when comparing unperturbed to perturbed data.

	Unperturbed (actual) data			Perturbed data		
**Gene name**	**Nominal p-value**	**BH corrected p-value**	**Rank**	**Nominal p-value**	**BH corrected p-value**	**Rank**
**SIGLEC5**	0.00181	0.21562	47	0	0	9
**CDC26**	0.00019	0.05328	20	0.00011	0.03247	19
**CXCL1**	0.00030	0.07537	22	0.00017	0.04307	21
**ZNF880**	0.00018	0.05217	19	0.00017	0.04307	22
**SPP1 ***	0.00014	0.04618	16	0.00022	0.05479	23

All five cd-ASE genes that have a different cd-ASE call when introducing an artificial mapping bias. BH, Benjamini-Hochberg. Rank, the rank of the gene in the meta-analysis. (*) SPP1 was the only gene that was present in the unperturbed set, but absent from the perturbed set; the other four genes in the table were present in the perturbed set, but absent from the unperturbed set.

Table S10. All 22 variants selected for validation. This table includes detailed raw data in terms of read counts and real-time quantitative RT-PCR CT-values for all alleles.

See separate file Table_S9.Edsgard_et_al.2015.xls.

Table S11. Number of detected ASE genes.

		**Static**		**cd-ASE**	
**Method**	*mode*	**ind**	**ind+meta**	**ind**	**ind+meta**
GeneiASE	regular	935	1389	11	19
GeneiASE	comparison	-	-	8	11
Intersection/Union (ratio)	*GeniASE comparison vs MBASED*	*812/2585 (0.31)*	-	*7/9 (0.78)*	-
Intersection/Union (ratio)	*GeneiASE regular+meta vs MBASED*				*7/20 (0.35)*
MBASED	comparison	2462	-	8	-

ind, individual; ind+meta, union of individual and meta-analysis across all 16 samples (static) or 8 individuals (cd-ASE). Regular (also called filtered) mode: GeneiASE static ASE run with no extra conditions, while GeneiASE cd-ASE was run pre-filtered on GeneiASE static ASE results – these two ways of running reflect the way the GeneiASE results and evaluation are presented in the manuscript. Comparison mode: no pre-filtering for cd-ASE; and results only include genes with at most 10 SNPs, since MBASED two-sample analysis stalled at genes with many SNPs. Intersection/union: intersection between and union of GeneiASE and MBASED results, with the fraction intersection/union within parenthesis. na: not applicable. MBASED was run in unphased mode since phase information is unavailable for these data.

Table S12. Phased versus unphased detection of genes with ASE.

		**Number of genes**		
**Method**	*mode*	**Genes w. ASE**	**Overlap with phased**	**Haplotype swapping**
GeneiASE (unphased)	*static*	59	46 (78.0%)	3 (5.1%)
MBASED - unphased	*one-sample*	85	68 (80.0%)	9 (10.6%)
MBASED - phased	*one-sample*	79	n.a.	0 (0%)

"Genes w. ASE": number of genes exhibiting significant ASE. "Overlap. with phased": number of ASE genes that overlap with phase-aware determination of ASE. "Haplotype swapping": number of genes exhibiting ASE towards both haplotypes (which means that the direction of the ASE changes within a gene, such that different variants exhibit ASE biased towards different haplotypes). All numbers pertain to genes with at least two variants. Data are from RNA-sequencing of the HapMap individual NA12878 where phasing is available.

Table S13. The list of detected ASE genes from NA12878.
The results include the results from GeneiASE, MBASED-unphased, and MBASED-phased. All numbers pertain to genes with at least two variants. Data are from RNA-sequencing of the HapMap individual NA12878 where phasing is available.

See separate file Table_S13.Edsgard_et_al.2015.xls

Table S14. All 3 cd-ASE genes with only one variant detected by GeneiASE.
The results include the findings from meta-analysis across the individuals.

See separate file Table_S14.Edsgard_et_al.2015.xls.

Table S15. All 693 static ASE genes with only one variant detected by GeneiASE.
The results include the findings from meta-analysis across the individuals.

See separate file Table_S15.Edsgard_et_al.2015.xls.

SUPPLEMENTARY METHODS AND RESULTS

Sample description

Power analysis was performed to determine the appropriate number of individuals to be used for RNA-sequencing, and eight individuals were concluded to give sufficient depth given the sequencing constraints (described below). Eight volunteers (four males, four females) giving informed consent were recruited in line with ethical approvals (2009/1374-32). The average age was 35 (27-47). Peripheral blood was extracted and white blood cell fractions separated to be subsequently treated with lipopolysaccharide (LPS) of Escherichia coli O55:B5 (Sigma Chemical Company, MO, USA). Cells from each volunteer were incubated at a concentration of 1 x 10^6 cells/ml in RPMI medium containing 10% FBS, 100 units/ml penicillin, and 100 µg/ml streptomycin, and treated with 1µg/ml LPS or left untreated for 12 hours at 37°C, 5% CO_2. Note that for each individual, untreated sample was kept and also used in the analysis, enabling a comparison of treated and untreated samples.

Genotyping

DNA was extracted from peripheral blood using the DNeasy Blood & Tissue Kit (Qiagen) and quantified using PicoGreen dsDNA Reagent according to manufacturer's recommendations (Invitrogen, Carlsbad, CA, USA). Genotyping was performed at the SNP&SEQ technology platform at Uppsala University (Sweden) using the Illumina Omni 2.5M SNP-arrays according to standard protocols. SNPs with a minor allele count of at least one were extracted and filtered requiring a 100% genotyping rate using plink (v.1.07), and converted to VCF (Variant Call Format) files using plinkseq (v.0.07).

RNA-sequencing and RNA-seq variant calling

Total mRNA from LPS-treated and untreated white blood cell fractions was extracted using Trizol® reagent (Invitrogen) and purified using the RNeasy Mini Kit following the manufacturer's instructions (Qiagen). Paired-end libraries were created according to standard protocols (Illumina Inc., San Diego, CA). Libraries were sequenced using Illumina HiSeq 2000 generating 2 x 100bp reads at the SNP&SEQ technology platform, Uppsala University, Sweden, according to the manufacturer's protocol using one Illumina HiSeq2000 flow-cell.

Before mapping, quality control was performed by 3'-trimming of reads, removing poly-A and poly-T tails as well as bases with a Phred score encoded by "B", which is an Illumina 1.5+ read segment quality control indicator indicating that the read end should not be used in further analyses. If a read after trimming was less than 40 bases long it was removed. Further, reads having five or more bases with a Phred score of 10 or lower, or ten or more bases with a Phred score of 20 or lower, were discarded. In addition, reads with four or more uncalled bases were also discarded. Overall quality after quality control filtering was verified by manual inspection of FastQC reports (http://www.bioinformatics.bbsrc.ac.uk/projects/fastqc).

Reads were mapped using TopHat (v.1.2.0; [4] to the hg19 reference genome using mate-distances from experimentally determined insert size distributions, exon annotations from Ensembl (v.59), and otherwise default parameters. Thereafter PCR duplicates were removed using Picard MarkDuplicates (v.1.41, http://sourceforge.net/projects/picard). Coverage was calculated using BEDTools (v.2.11.2; [5]).

RNA-seq variant calling was performed using SAMtools mpileup (v.0.1.18) adjusting the max per-sample depth from 250 to 10000 to handle highly expressed regions and setting the minimum mapping quality (-q) to 1 to remove non-uniquely mapped reads. Variants were called for each individual, using the information from all samples.

Annotation of variants

Annotation of variants was done using Annovar [6] (v.2011.05.06) and custom perl and R scripts. To link variants showing ASE to differentially expressed genes, variants annotated with a HUGO gene symbol were associated with the corresponding differential expression of the gene.

Differential expression analysis

Read counts were obtained using htseqcount (v.0.5.1) and were based on Ensembl gene annotations (Ensembl v.59). We used the default parameters of htseqcount apart from "--stranded=no" to specify that we did not use a strand-specific protocol. Subsequently, differential expression analysis was performed using the R package

DESeq v.1.4.1 [7]. Pathway enrichment of differentially expressed genes was done using a hypergeometric test on gene sets retrieved from KEGG [8], Reactome [9], BioCarta (http://www.biocarta.com), NCI-Nature curated pathways [10], GO [11], COSMIC [12], Cyclebase [13], protein-protein interaction complexes [14], OMIM (Online Mendelian Inheritance in Man, http://www.ncbi.nlm.nih.gov/omim) and MGI (Mouse Genome Informatics, http://www.informatics.jax.org). Terms annotating more than 700 or less than five genes were excluded, since they do not produce meaningful statistical results.

We used DESeq [7] (v.1.4.1) to perform a differential expression analysis of the group of LPS treated samples versus the group of untreated samples. Out of 35,215 Ensembl genes, 5,395 (15.3%) were significantly differentially expressed (adjusted $P < 0.05$). Pathway analysis of the top 250 differentially expressed genes resulted in 165 significantly enriched terms, all related to immune response, including "response to lipopolysaccharide" (hypergeometric test, P = 7.2*10-8). In a principal component analysis of FPKM values (Fragments Per Kilobase of exon per Million fragments mapped), all 16 samples clustered in agreement with their condition (treated/untreated), apart from one of the LPS treated samples which showed a tendency of being an outlier (Supplemental Fig. S3). These results indicated a high quality of our data since LPS was intentionally used to induce an inflammatory response.

Power analysis for allele-specific expression detection

To estimate the sequence depth required to detect an allelic imbalance at different allelic ratios we performed power calculations. They showed that at a sequence depth of 50, a 2-fold difference in expression (67:33), and a significance level of P=0.001, the sensitivity is 19% (Fig. S13). The nominal significance level was chosen based on the assumption that we observe approximately 5000 heterozygous SNPs within a single sample with sequence depth above 50, and where 5% of these have a P-value less than 0.001, resulting in a multiple testing corrected false discovery rate (FDR) of ~1%. These assumptions were based on the findings in Heap et al. 2010 [15]. Another study identified ~1500 heterozygote SNPs per individual, but using a read depth down to 6 [16]. Paired end sequencing using a whole flowcell (8 lanes) with Illumina HiSeq 2000 was expected to yield approximately 100Gb of sequence according to

manufacturers specifications. Sequencing 16 samples was expected to result in an average sequence depth of the transcriptome of 85 before mapping and QC (100Gb / (16 samples * 70Mb)), where the transcriptome size (70Mb) was calculated from the UCSC hg19 RefSeq genes. Assuming that 59% of bases are retained after mapping and QC (Montgomery et al. 2010, who performed RNA-seq on 60 CEU individuals retained 56% on average per sample) would result in an average sequence depth above 50 if sequencing 16 samples. The vast majority of genes were anticipated to have a coverage below the average coverage, since the coverage per SNP approximately follows an exponential distribution [17]. The expression of biologically relevant transcripts was however increased by activating an immune response by treating the cells with lipopolysaccharide (LPS). The genotype calling of heterozygous SNPs from the RNA-seq data was expected to be highly reliable at a coverage of 50. Simulations estimate that 97% of heterozygous genotypes are correctly inferred at an allelic imbalance of 80:20 [17]. Retrospectively we observed that a depth of 10 was sufficient to find a high number of significant variants exhibiting ASE after multiple testing correction in the static ASE analysis. This may in part be due to overly conservative assumptions in the *a priori* power analysis.

Reference mapping bias

An inherent problem in assessing static ASE using RNA-seq data is that the read mapping will be biased towards preferably mapping alleles identical to the reference genome, whereas reads differing from the reference genome will have a lower mapping quality or too many mismatches and thereby be at higher risk of being discarded. This could be resolved by mapping to a diploid genome if the individual's diploid genome sequence is available [18,19], but often only RNA-seq data for a single familial representative is available, thus prompting the use of a reference genome for read mapping. For analysis of condition-dependent ASE this is less of an issue, since most of the mapping bias is cancelled out when comparing two conditions. However, for static ASE analysis where ASE, and not change of ASE, is to be detected, it remains a major issue. A number of approaches have been suggested to remedy the mapping bias issue given RNA-seq data, e.g. changing the expected allelic ratio, 0.5, to the mapping ratio of simulated reads with equal allelic ratio (Montgomery et al. 2010); mapping reads to an individual-specific transcriptome reference generated from phasing of RNA genotype calls of the individual [20]; and others [21], Heap et al.

2010, [22]. It is yet unclear which approach is best and we therefore evaluated two methods to reduce the mapping bias, that of Montgomery et al and that of Turro et al. To evaluate the mapping bias we simulated read data with equal allelic ratio for all observed heterozygous variants in the same manner as Degner et al. (2009) [21], and observed a mapping bias for approximately 5% of the variants with significant ASE. We stratified the significant variants into those called as heterozygous by both the SNP-array and RNA-seq, by SNP-array only, and by RNA-seq only, and we observed mapping bias for 0.0%, 0.2%, and 10.3%, respectively, of these variants. To correct for the mapping bias we used the estimated bias from the simulated mapping ratios in a modified binomial test [16]. Mapping bias was reduced but not eliminated by this method. We applied MMSEQ [20], which phases called variants to construct individual-specific transcriptomes against which read mapping is performed. In our 16 samples, approximately 9% of variants per sample were successfully phased, resulting in a total of 4,329 phased unique variants, in 6,574 transcripts and 966 genes. ASE analysis retrieved 426 phased variants with significant ASE. Mapping bias was completely removed from the 4,329 phased variants and, accordingly, also from the 426 variants exhibiting ASE.

Synthetic data generation

(i) Synthetic data for assessing FDR of the empirical data set

We generated a synthetic RNA-seq data set comprising 16 samples, with parameters sampled from our real data. The synthetic data was analyzed identically to the real data as to estimate FDRs at SNV level. SNVs from the 1000 Genomes Project (TGP) were downloaded (November, 2010, release) and all heterozygous SNVs in the CEU population that had a consensus genotype from at least two of the four TGP sequencing centers were extracted [23]. Exonic variants were extracted using Ensembl annotation (v.64). Synthetic haplotypes were constructed by binomial sampling of alleles based on the minor allele frequency (MAF) in the CEU population. ASE levels of the haplo-isoforms were sampled from the expression distribution estimated by MMSEQ from the real RNA-seq data in this study, thus using haplotype information for reads spanning more than one SNV. Given the synthetic haplotypes and expression levels, paired-end reads were simulated using maqsim (MAQ [24]; v. 0.7.1). Insert sizes from the real dataset were used and the base quality distribution was sampled from real data using maq simutrain (MAQ 0.7.1). Base qualities were

converted between phred+33 and phred+64 encoding using ill2sanger (MAQ 0.7.1) and seqret (EMBOSS [25]; v. 6.4.0). FDRs were calculated by employing the same analysis on the synthetic reads as was done for the real data, including QC, read mapping, variation calling, and ASE analysis. The resulting significant variants were then compared to the set of true positives resulting from ASE analysis of the synthetic allele fractions.

(ii) Synthetic data for assessing GeneiASE performance at varying noise levels, effect sizes, and read depths

To estimate the performance with respect to different properties of the input count data we generated synthetic data sets varying the read depth, effect size and noise-level. The effect size used for static ASE was the alternative allelic fraction, p = alt reads / (alt + ref reads), and it was varied from 0.55 to 0.8. The effect size used for icd-ASE was the odds-ratio and it was varied from 1.1 to 16. Read-depths, reflecting the sum of the read counts at the alternative and reference allele of a variant, were varied from 10 to 100. We let the noise-level reflect the varying degree of ASE of different variants within a gene that is technical, non-biological, variation (see "random effect model" property, P3, below). We used the log-odds as a measure of the ASE effect and modelled it with a normal distribution, adding noise from ~N(0, sd), where sd thereby reflected the noise-level. Using DNA data we estimated the true noise level to sd = 0.22.

The effect of alternative splicing on mapping bias in cd-ASE

We have identified one rare exception where the mapping bias is not cancelled out in cd-ASE analysis. This exception can arise in the event of alternative splicing when an exon with a heterozygous variant is expressed in both conditions while a nearby exon is expressed in only one of the conditions, and where that nearby exon contains a region within read length distance from the heterozygous variant, and where this region also has such properties so as to introduce mapping bias. This mapping bias would then affect the read counts for all coordinates within read length distance. Thus, alternative splicing might generate false positives for a small set of genes where all these conditions are satisfied. The sensitivity (false negative rate) of cd-ASE analysis can, however, be affected due to a reduced number of reads stemming from

mapping bias, and the direction of ASE can be reversed in extreme cases. For instance, consider the case where the actual expression level of the alternative allele is higher than the reference allele in the treated state, but the expression levels of the two alleles are equal in the untreated state. Mapping bias against the alternative allele could then cause the alternative allele to appear as being lower expressed (than the reference allele) in the untreated state and the two alleles being expressed at equal levels in the treated state. However, since this is a systematic shift across the two conditions, the difference in ASE between the two conditions, $\Delta ASE_{RNA\text{-}seq}$(T-U), would be the same as if mapping bias was not present.

Using GeneiASE for ASE detection in single variant genes

In the main text, we used GeneiASE with filters that included the requirement that two dbSNP variants should be present. This setting precludes GeneiASE to detect genes with only a single variant. For these genes, we instead relied on Fisher's exact test for cd-ASE and a modified binomial test for static ASE. There were two main reasons for this: (i) To facilitate comparison of the number of variants and genes that exhibit ASE in our particular dataset as compared to datasets in previous studies, which have used such (more conventional) approaches; and (ii) To provide baseline reference results using previously accepted methods[16,21], against which the novel GeneiASE results could be compared. However, we also ran GeneiASE focusing on genes with only a single variant, resulting in 3 cd-ASE and 693 static ASE genes. These results are shown in Supplemental Tables S14 (cd-ASE) and S15 (static).

In the two approaches outlined above (GeneiASE vs. Fisher's exact test/modified binomial test), we also used two different read count models: modified binomial for variant ASE detection, and beta-binomial for GeneiASE gene level detection. These models are actually not as different as it first may seem. Using the DNA allelic information in the GeneiASE construction, we attempted to account for technical variation as well as sequence specific bias, and we modeled this with the overdispersion parameter of the beta-binomial. In the single-variant analysis, we handled potential reference mapping bias by simulating data as in Degner et al[21] and adjusting the null-hypothesis of the binomial. This in effect renders a similar model since it will cause each variant to have a different null-p, which is exactly what the

beta-binomial models imply (a varying p, sampled from an underlying stochastic distribution).

The relationship between GeneiASE and other methods

We identified six properties, P.1-P.6, that a well-powered model should incorporate to identify transcripts with condition-dependent allele-specific expression (cd-ASE) from RNA-seq data for individuals where the diploid genome is unavailable (unphased data). P.1) Paired model, since the data is from the same individual under the different treatment conditions. P.2) Binomial model, rather than a Poisson, since the marginal sum of the counts from two alleles is fixed. P.3) Random effect model, since the effect may vary along a gene (for different variants), due to for example technical noise. P 4) Variance stabilization of effect sizes. P.5) Estimation of the null model from DNA. P.6) Independence of the effect directionality between variants, since unphased data. Several of these properties are also valid for detection of static ASE.

To our knowledge there is no method that features all six properties, which motivated us to design a method that can be used to detect cd-ASE. In particular, most methods designed to analyze similar problems would need to be modified as to be able to accommodate unphased data (P.6). Below we list a number of methods that handles similar problems and discuss their drawbacks with respect to testing for cd-ASE. A comparison between these methods with respect to the five properties listed above is summarized in Supplemental Table S5.

The read count data from two alleles under two treatments can be represented by k 2x2 tables, where k is the number of variants (strata). Two classical meta-analysis methods to analyze such data is the Mantel-Haenszel method (MH) [1] and DerSimonian-Laird (DL) [2]. MH is a fixed effect model where the null hypothesis is that there is no association in any stratum, and that the counts in each stratum (each 2x2 table) follows a hypergeometric distribution. The association effect, β, can be calculated as the log-odds ratio, which under the null hypothesis of a hypergeometric distribution is normally distributed with variance σ^2, where σ^2 is the sampling error. Drawbacks with this method is: first, the observed counts will not follow a hypergeometric, since there is noise in the data (P.3), second, even if the noise would be neglible the effect will not be normally distributed, but rather follow a half-normal,

since we take the absolute value of the effect to handle unphased data (P.6). The model of DerSimonian-Laird do account for a random effect, where the estimated effects are assumed to follow, $\beta_j = \beta_j + \varepsilon_j \widehat{\beta_j} = \beta_j + \varepsilon_j$, where β_j is the true effect, with variance τ_j^2, and ε_j is a normal variate with variance σ^2 reflecting the standard error. However, the heterogeneity in effect between SNVs, encoded by τ, is typically estimated from observed counts within a single gene, which makes the estimation worse than if using whole-genome DNA data (P.5). Second, DL does not handle unphased data (P.6).

Another meta-analytical approach is to combine a set of p-values. Two common methods are those of Fisher and Liptak-Stouffer (LS). In Fisher's method the p-values are multiplied, and -2*log of the product has a central χ^2 distribution under the null-hypothesis. In Liptak-Stouffer each p-value is converted to a Z-score and summed up, $LS = \sum\Phi^{-1}(1 - p_j)/\sqrt{k}$, which has a unit normal distribution under the null-hypothesis. Multiplication of the p-values implies a null hypothesis that there is no association in any stratum, whereas the addition in LS implies a tendency to require an effect in several strata. We applied LS using Fisher's test for each 2x2 table. We observed lower power and a conservative non-uniform p-value distribution for this approach as compared to GeneiASE, which is likely due to two reasons. First, Fisher's method assumes a hypergeometric distribution, and does therefor not take over-dispersion (noise) into account (P.3). Second, even when setting the noise to zero in our simulations, lower power and non-uniform p-value distributions were still observed. This is due to the discreteness of the data, and in fact Fisher's test is known to be conservative even at relatively high sample sizes such as 1000 [26]. This effect is further exacerbated when combining the results from several Fisher's test.

More recent methods which have been applied to problems similar to that of identifying cd-ASE using RNA-seq data, include a Bayesian beta-binomial model [27], MMSEQ [20], MBASED (Mayba et al. 2014), and an inverted beta model [28].

Skelly's method is designed to identify ASE in samples for which the diploid genome is known (P.6). We attempted to run Skelly's method in a modified manner by setting the ASE to be in the same direction for all SNVs within a gene, but did not succeed in obtaining reasonable results. Even if one would make a modification to Skelly's method that could handle unphased data, the test is not paired (P.1) and one would

therefor only be able to test changes from no significant ASE in one condition to significant ASE in the other, or vice versa.

MMSEQ is a pipeline that infers the expression levels of the isoforms from each haplotype, so called haplo-isoforms, and it relies on phasing the data as to infer the haplotype. We ran the method by using the genotype calls from the RNA-seq data as input to the phasing procedure, which was part of the MMSEQ pipeline, but only a few percent of all variants were successfully phased. With better phasing the method may have worked but the phasing is likely to contain many errors as long as no DNA is available (P.6).

Pham and coworkers [28] designed a paired sample test for count data using an inverted beta model. Their model is intended to identify a treatment effect on the total expression level (differential expression) in a paired experimental design given a set of individuals. Since they model total expression of a protein (or transcript) rather than allele-specific expression they let the observed counts of a transcript be Poisson distributed, each with a parameter π_i*t, where t is the total number of reads from a sample. The treatment effect is specified as a quota between the effects from the treatment groups, $\phi = \pi_{it} / \pi_{iu}$, and they introduce random effects (variation in effect between individuals) by letting ϕ be a random variable generated from an inverted beta distribution. Two drawbacks of this model with respect to applying it to cd-ASE is that they use a Poisson distribution rather than a binomial distribution (P.2) and, more importantly, that it assumes that the effect in different strata is in the same direction (P.6).

The MBASED method (Mayba et al. 2014) is presented in the setting of ASE in cancer tissues and cell lines, and is possible to run both in one-sample and two-sample modes, corresponding to our static and cd-ASE modes, respectively. Thus, their method deals with paired samples (P.1). It relies on a pseudo-phasing of the RNA-seq data (P.6) if provided data are not phased, and builds on combining ASE scores derived from the major/minor allele frequencies for multiple SNPs within a gene in a sample (P.3). In two-sample mode, the two samples are treated in an asymmetrical way, such that the phasing is transferred from the reference sample to the other sample (it is of course possible to swap reference and other). The background is estimated from the RNA-seq data and for their two-sample analysis they do not

perform a variance stabilization of the effect sizes although this is performed for their one-sample analysis (i.e., P.4 and P.5 not fulfilled).

Using our data to compare with other ASE detection methods
Skelly's method [3], MMSEQ [20], and MBASED [29] v1.2.0 were downloaded and run locally according to instructions, and tested on the RNA-seq data in our study (LPS+/-; 8 individuals; unphased).

The MMSEQ pipeline (v. 0.9.18) was applied with some modifications. Reads were first subjected to quality control, and duplicates were removed after read mapping (Supplemental Methods). Due to the large size of the dataset the pipeline needed to be run on many CPU's in parallel and necessary amendments of the MMSEQ pipeline were performed to this end, in particular the steps related to the phasing of variants.

MBASED was tested in both one-sample and two-sample modes, including, as for GeneiASE, genes with >1 variant. The methods were run on a powerful 128-core 512 Gb RAM shared-memory Linux server. MBASED in one-sample mode worked without any issues in our testing, however, running MBASED in two-sample mode on our data set, it was far from finishing after seven days, despite running it in parallel on a 128-core 512Gb 64-bit shared-memory Linux server. We back-traced the problem to genes with many SNPs. For example, using 10 bootstrap-samplings and inputting a single gene from a single individual, genes with less than five SNPs finished in less than one second, whereas a gene with 19 SNPs took 9.3 minutes. Given that the recommended bootstrap-size by the MBASED authors is 100,000, and since the computational complexity is linear, we concluded that the reason that MBASED program stalled on our data set was that it contained genes with many SNPs. To ensure that it was not a version or package dependency issue, we ran both the latest version of MBASED (v1.2.0 under R 3.2.0) as well as an older version (v1.0.0 under R 3.1.0) on two different computers. To make the comparison with GeneiASE as fair as possible on the real data set, a (non-optimal) GeneiASE cd-ASE "comparison mode" was constructed, where any genes with more than 10 SNPs were removed, and the static-ASE gene filtering was not applied. The results of the comparison between GeneiASE and MBASED are presented in Supplementary Table S11.

GeneiASE was also compared against more simplistic approaches, using either the binomial exact test, where the null hypothesis of the mean was adjusted for mapping bias (for static ASE, Fig. S10), or Fisher's exact test (for icd-ASE, Fig. S11), in combination with Stouffer's method. Briefly, p-values were calculated for each SNV within a gene. The p-values were transformed to Z-scores by the inverse of the normal distribution and the Z-scores were combined by Stouffer's method [3]: $Z_{pooled} = \sum \Phi^{-1}(1 - p_j)/\sqrt{k}$. The p-value was then obtained from one minus the quantile of the normal distribution at the pooled Z-score value.

Mapping-bias influence on GeneiASE cd-ASE gene level results

We tested whether GeneiASE cd-ASE detection was robust with respect to mapping bias by perturbing the read counts of our LPS treated and untreated data sets. The perturbation was performed such that the reference allele read count was increased and the alternative allele count was decreased (see below). In effect, this means that we construct an artificial mapping bias in our data. We performed GeneiASE cd-ASE analysis to retrieve significant genes, in the same manner as for the unperturbed empirical data, including the meta-analysis. From DNA-data we have estimated the variability of measured ASE between variants. Since this variability is partly due to varying mapping bias between different loci we considered it reasonable to use a degree of perturbation similar to this variability in studying the effect that mapping biases may have on GeneiASE meta-analysis results. The log-odds of the ASE distribution approximately follows a normal distribution, we therefore fitted a normal distribution to the DNA data, observing a standard deviation of 0.22. This corresponds to a change of the ASE of 0.05, where the ASE is defined as the fraction of alternative allele read counts, p = n.alt / (n.alt + n.ref). The perturbation given the log-odds is derived from the logistic equation, p = logit(log.odds) = 1 / (1 + exp(-log.odds)). We then perturbed the read counts at each variant such that, p.perturbed = p - 0.05, that is, reducing the alternative allele counts and increasing the reference allele counts. We kept the read sum fixed, since otherwise the power would be affected. 1.2% of the variants would get a negative value by this, since they had very low or zero read count, and was therefore not perturbed. We note that perturbing 98.8% of the variants is still a greater perturbation than what would be expected from sequence-specific mapping bias and we are therefore not underestimating its effect.

At the individual level, GeneiASE detected 14 genes in the perturbed data, adding three additional genes to the 11 found in the unperturbed case. The three genes included *CD101*, *CNOT2*, and *SIGLE5C*. All three of these genes had multiple-testing adjusted p-values in the range 0.05-0.06 in the unperturbed case and were therefore borderline significant with respect to a significance level 0.05. Furthermore, *CD101* and *CNOT2* were picked up in the meta-analysis of the unperturbed data.

With respect to meta-analysis across individuals we found 22 genes in the perturbed data as compared to the 19 genes for the unperturbed data presented in the results of the main text, where four were unique to the perturbed set and one to the unperturbed. Three of the four additional genes found in the perturbed data were borderline significant in the unperturbed data with p-values 0.052, 0.053 and 0.075, whereas the fourth gene had a p-value of 0.21; however, this fourth gene was *SIGLE5C* which was borderline significant at an individual-level in the unperturbed data. *SPP1* was the one gene that was lost when introducing artificial mapping bias. In summary, even though we use a perturbation that is stronger than one would expect to be present due to mapping bias, very few additional genes pass the significance threshold, and those that do are already border-line significant in the original unperturbed data. The results are summarized in Supplementary Table S9.

Consistency of meta-cd-ASE with regards to individual variability

We checked the consistency with respect to the direction of the cd-ASE among variants that were carried by several individuals. In the 19 cd-ASE genes, there were 51 variants in 14 genes that were shared by more than one individual (column "n.individuals" in Supplementary Table S6). Four of the 51 variants had a cd-ASE direction that differed between at least two individuals. We considered cd-ASE direction to be different if the log-odds-ratio had a different sign and if its confidence interval did not overlap 0.

Phasing

We tested GeneiASE (static) and phased and unphased variants of MBASED (one-sample mode) on HapMap individual NA12878 for which phased data from a single

condition RNA-sequencing experiment is available. The results are presented in the main manuscript and in Supplementary Table S12.

Haplotype swapping: ASE that changes haplotypes within genes

To investigate how common it is that the ASE is changing haplotypes within genes (which means that the direction of the ASE changes within a gene, such that different variants exhibit ASE biased towards different haplotypes), we analyzed the NA12878 data from this perspective. We note that it is only relevant to assess this among significant genes, since in genes with no ASE, exhibiting a 50/50 expression ratio between the haplotypes, a varying direction is frequently observed due to sampling error. Furthermore, the sampling error is especially pronounced for variants with low read counts. Due to this, we took the read depth at a variant into account by calculating the confidence interval of the ASE for each variant using Wilson's method, and deemed a gene to have varying ASE (haplotype swapping) if it had at least two variants with ASE in the opposite direction and whose confidence intervals did not overlap 0.5. For GeneiASE, 5.1% (3/59) of the genes exhibited ASE towards both of the haplotypes, at different variants, while for unphased MBASED, 10.6% (9/85) of genes, exhibited varying direction of the ASE, (and none for phased MBASED). The results are summarized in Supplementary Table S12 and the full list of detected genes is in Supplemental Table S13.

Real-time quantitative RT-PCR validation

Validation of ASE was performed as previously described with minor modifications [30]. 500ng of mRNA from each sample were reversed transcribed using the SuperScript™ II reverse transcriptase enzyme and the synthesized cDNA was used for real-time quantitative RT-PCR validation of ASE candidates. The TaqMan® SNP Genotyping assay (pre-designed or customized) for each ASE candidate was mixed with TaqMan® Gene expression master mix (Applied Biosystems) to a final volume of 25 µl. Optimal reaction conditions were 95°C for 10 min and 40 cycles of 95°C for 15 s, 60°C for 60 s. The fluorescence emitted by the two alleles (VIC or FAM dye) was reported as cycle threshold (CT) value. Each sample was subjected to three

independent RT-PCR validations, yielding technical triplicates of each CT-value. The change of ASE between the two conditions in the RT-PCR experiments is defined as:

$\Delta ASE_{RT\text{-}PCR}(T\text{-}U) = ASE_{RT\text{-}PCR}(T) - ASE_{RT\text{-}PCR}(U)$,

where $ASE_{RT\text{-}PCR}$ is the difference in mean cycle threshold values between the two alleles for either condition (T or U). Supplementary Table S10 contains detailed raw data in terms of read counts and real-time quantitative RT-PCR CT-values for all alleles selected for validation.

SUPPLEMENTARY REFERENCES

1 Mantel, N. & Haenszel, W. Statistical aspects of the analysis of data from retrospective studies of disease. *Journal of the National Cancer Institute* **22**, 719-748 (1959).

2 DerSimonian, R. & Laird, N. Meta-analysis in clinical trials. *Controlled clinical trials* **7**, 177-188, (1986).

3 Liptak, T. On the combination of independent tests. *Magyar Tudomanyos Akademia Matematikai Kutato In-tezetenek Kozlemenyei*, (1958).

4 Trapnell, C., Pachter, L. & Salzberg, S. TopHat: discovering splice junctions with RNA-Seq. *Bioinformatics* **25**, 1105-1111, (2009).

5 Quinlan, A. & Hall, I. BEDTools: a flexible suite of utilities for comparing genomic features. *Bioinformatics (Oxford, England)* **26**, 841-842, (2010).

6 Wang, K., Li, M. & Hakonarson, H. ANNOVAR: functional annotation of genetic variants from high-throughput sequencing data. *Nucleic acids research* **38**, e164-e164, (2010).

7 Anders, S. & Huber, W. Differential expression analysis for sequence count data. *Genome Biology* **11**, R106, (2010).

8 Ogata, H. *et al.* KEGG: Kyoto Encyclopedia of Genes and Genomes. *Nucleic acids research* **27**, 29-34, (1999).

9 D'Eustachio, P. Reactome knowledgebase of human biological pathways and processes. *Methods in molecular biology (Clifton, N.J.)* **694**, 49-61, (2011).

10 Schaefer, C. *et al.* PID: the Pathway Interaction Database. *Nucleic acids research* **37**, D674-D679, (2009).

11 Ashburner, M. *et al.* Gene ontology: tool for the unification of biology. The Gene Ontology Consortium. *Nature genetics* **25**, 25-29, (2000).

12 Forbes, S. *et al.* COSMIC: mining complete cancer genomes in the Catalogue of Somatic Mutations in Cancer. *Nucleic acids research* **39**, (2011).

13 Gauthier, N., Jensen, L., Wernersson, R., Brunak, S. r. & Jensen, T. Cyclebase.org: version 2.0, an updated comprehensive, multi-species repository of cell cycle experiments and derived analysis results. *Nucleic Acids Research* **38**, D699-D702, (2010).

14 Lage, K. *et al.* A human phenome-interactome network of protein complexes implicated in genetic disorders. *Nature biotechnology* **25**, 309-316, (2007).

15 Heap, G. *et al.* Genome-wide analysis of allelic expression imbalance in human primary cells by high-throughput transcriptome resequencing. *Human Molecular Genetics* **19**, 122-134, (2010).

16 Montgomery, S. *et al.* Transcriptome genetics using second generation sequencing in a Caucasian population. *Nature* **464**, 773-777, (2010).

17 Nothnagel, M. *et al.* Statistical inference of allelic imbalance from transcriptome data. *Human mutation* **32**, 98-106, (2011).

18 Rozowsky, J. *et al.* AlleleSeq: analysis of allele-specific expression and binding in a network framework. *Molecular systems biology* **7**, (2011).

19 Chen, R. *et al.* Personal omics profiling reveals dynamic molecular and medical phenotypes. *Cell* **148**, 1293-1307, (2012).

20 Turro, E. *et al.* Haplotype and isoform specific expression estimation using multi-mapping RNA-seq reads. *Genome Biology* **12**, R13, (2011).

21 Degner, J. *et al.* Effect of read-mapping biases on detecting allele-specific expression from RNA-sequencing data. *Bioinformatics* **25**, 3207-3212, (2009).

22 Vijaya Satya, R., Zavaljevski, N. & Reifman, J. A new strategy to reduce allelic bias in RNA-Seq readmapping. *Nucleic Acids Research*, (2012).

23 1000 Genomes Project Consortium. A map of human genome variation from population-scale sequencing. *Nature* **467**, 1061-1073, (2010).

24 Li, H., Ruan, J. & Durbin, R. Mapping short DNA sequencing reads and calling variants using mapping quality scores. *Genome Research* **18**, 1851-1858, (2008).

25 Rice, P., Longden, I. & Bleasby, A. EMBOSS: the European Molecular Biology Open Software Suite. *Trends in genetics : TIG* **16**, 276-277, (2000).

26 Rohlfs, R. V. & Weir, B. S. Distributions of Hardy-Weinberg equilibrium test statistics. *Genetics* **180**, 1609-1616, (2008).

27 Skelly, D., Johansson, M., Madeoy, J., Wakefield, J. & Akey, J. A powerful and flexible statistical framework for testing hypotheses of allele-specific gene expression from RNA-seq data. *Genome Research* **21**, 1728-1737, (2011).

28 Pham, T. & Jimenez, C. An accurate paired sample test for count data. *Bioinformatics (Oxford, England)* **28**, (2012).

29 Mayba, O. *et al.* MBASED: allele-specific expression detection in cancer tissues and cell lines. *Genome Biol* **15**, 405, (2014).

30 Chen, X. *et al.* Allelic imbalance in BRCA1 and BRCA2 gene expression is associated with an increased breast cancer risk. *Human molecular genetics* **17**, 1336-1348, (2008).
